# High Oxidation State V‐O‐Co Structure Promotes the Role of V‐^*^O Intermediates in Efficient HMF Oxidation

**DOI:** 10.1002/advs.202508912

**Published:** 2025-08-13

**Authors:** Honglei Wang, Xue Wen, Jiuxiang Dai, Dawei Xu, Xianzhu Luo, Pan Luo, Hongshuai Cao

**Affiliations:** ^1^ College of Chemical Engineering and Technology Key Laboratory of Catalytic Conversion Energy Coupling in Shanxi Province Taiyuan University of Science and Technology Taiyuan 030024 China; ^2^ School of Chemistry and Chemical Engineering School of Electronics Information and Electrical Engineering Shanghai Jiao Tong University Shanghai 200240 China; ^3^ Key Laboratory of Hainan Trauma and Disaster Rescue, Department of Wound Repair, The First Affiliated Hospital of Hainan Medical University, Hainan Medical University, Key Laboratory of Hainan Functional Materials and Molecular Imaging, College of Emergency and Trauma Hainan Medical University Haikou 571199 China; ^4^ Department of Plastic Surgery Beijing Chaoyang Hospital Affiliated to Capital Medical University Beijing China

**Keywords:** efficient HMF oxidation, high oxidation state, multi‐substrate catalysis, universal catalytic intermediate, V‐^*^O intermediates

## Abstract

Designing heterometallic oxygen‐bridge structure (M1‐O‐M2) to achieve the efficient and stable electrochemical conversion of 5‐hydroxymethylfurfural (HMF) to 2,5‐furandicarboxylic acid (FDCA) has become an important approach to address the rapid consumption of fossil fuels and mitigate white pollution. However, designing effective M1‐O‐M2 structures and elucidating their catalytic mechanisms remains a key challenge in this field. In this study, a high oxidation state V‐O‐Co structure is constructed by doping V into reconstructed CoOOH, which promotes the formation of the active intermediate V‐^*^O. The catalyst achieves a low HMF oxidation onset potential of 1.13 V vs. RHE, the HMF conversion rate reaches 99.6%, the FDCA selectivity reaches 97.8%, and the Faradaic efficiency reaches 97.0% at an applied potential of 1.30 V vs. RHE. Based on this novel design strategy, V‐doped Ni_2_P/NF is further constructed, which also forms the active intermediate V‐^*^O, validating the effectiveness of this strategy. Notably, the oxidation state‐controlled V‐O‐Co structure demonstrates excellent catalytic performance in promoting the efficient conversion of various organic compounds (such as glycerol, glucose, methanol, etc.) into high‐value chemicals, providing a precedent for the development of single hetero‐oxygen‐bridge structures for the effective conversion of various high‐value compounds.

## Graphical abstract

By designing a high oxidation state V_H_‐O‐Co_H_ structure, the V‐^*^O intermediate can be effectively generated, thereby facilitating the catalytic oxidation of HMF. This innovative design strategy demonstrates broad applicability, as it not only enables the formation of the active intermediate in various materials (V‐Ni_2_P), but also facilitates its catalytic conversion of multiple organic substrates, including glycerol, glucose, and methanol.

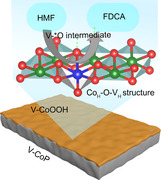

## 1 Introduction

The electrocatalytic conversion of biomass into high‐value chemicals and biodegradable polymers is of great significance for reducing dependence on fossil fuels and alleviating white pollution.^[^ ^1^ ^]^ In particular, 2,5‐furandicarboxylic acid (FDCA), which is prepared through the electrocatalytic oxidation of 5‐hydroxymethylfurfural (HMF), has attracted widespread attention due to its rigid furan ring structure, excellent mechanical properties, good thermal stability, and potential applications in the synthesis of specialty functional polymers.^[^ ^2^ ^]^ However, the electrocatalytic oxidation of HMF involves multiple proton‐coupled electron transfer steps and the formation of complex intermediates, leading to low reaction concentrations and high energy consumption. Specifically, the development of efficient electrocatalysts to achieve low energy consumption (e.g., with an overpotential below 1.2 V at a current density of 10.0 mA cm^−2^) while ensuring high conversion rates remains a key challenge. Furthermore, the HMF concentrations reported in the literature for electrocatalytic reactions are generally lower than 100.0 mm, and the yield is below 300 mg h^−1^.^[^ ^3^ ^]^ While low concentrations can effectively reduce side reactions and improve catalyst selectivity, maintaining selectivity at higher concentrations remains a challenge in research. Therefore, the development of efficient and stable anode materials to promote HMF catalysis has become an urgent task. Previous design strategies for anode materials have primarily focused on the development of ∜cheap∝ electrocatalytic materials based on transition metals, which accelerate the catalytic reaction kinetics by restructuring active species.^[^ ^4^ ^]^ Despite some progress in improving conversion efficiency, the efficient and sustained conversion of HMF remains an unresolved issue, particularly due to the lack of in‐depth understanding and research into the design of active intermediates and the catalytic mechanism of HMF.

The heterometallic oxygen‐bridge structure (M1‐O‐M2), commonly found in bimetallic transition metal‐based electrocatalyst materials, connects different metal elements through an oxygen bridge, forming catalytically active sites with synergistic effects. During the electrocatalytic process, this structure facilitates electron transfer, provides diverse active sites, and regulates the adsorption behavior of intermediates, thereby significantly improving catalytic efficiency and selectivity.^[^ ^5^ ^]^ It has been widely applied in enhancing the activity of the oxygen evolution reaction (OER),^[^ ^6^ ^]^ efficiently degrading organic amines,^[^ ^7^ ^]^ and promoting the catalytic oxidation of HMF.^[^ ^8^ ^]^ In these applications, the metal centers of M1 and M2 are usually in different oxidation states, and the difference in their valence states helps promote the efficient conversion of HMF.^[^ ^9^ ^]^ Therefore, constructing an oxidation state‐regulated M1‐O‐M2 structure could help solve the above challenges. Unfortunately, it is difficult to construct an oxidation state‐regulated M1‐O‐M2 structure through the reconstruction of active species, which severely limits the further application of bimetallic transition metal‐based electrocatalyst materials in HMF conversion.

Whether following the AEM (adsorbate evolution mechanism) or LOM (lattice oxygen oxidation mechanism), the intermediates generated by the reconstruction of active species in OER catalysis (such as ^*^OH and ^*^O) play a crucial role in the electrooxidation of organic compounds, particularly under alkaline conditions.^[^ ^10^ ^]^ Numerous studies have explored the role of ^*^OH in HMF catalysis, where ^*^OH, as a mild oxidation intermediate, is primarily used for the selective oxidation of functional groups.^[^ ^11^ ^]^ Compared to M‐^*^OH, M‐^*^O has stronger oxidation properties, effectively promoting the cleavage of C‐H bonds and dehydrogenation reactions in HMF molecules, thereby increasing the generation of selective products and significantly accelerating the oxidation reaction rate. Furthermore, unlike M‐^*^OH, which easily undergoes competitive reactions with reactants, M‐^*^O enhances the stability of the catalyst surface, thereby improving the long‐term stability of the reaction.^[^ ^12^ ^]^ Based on this, designing a M1‐O‐M2 structure to promote the formation of highly oxidative ^*^O can enable efficient HMF conversion. Furthermore, using a single M1‐O‐M2 structure to form high‐oxidation ^*^O intermediates can facilitate the efficient conversion of various organic compounds, such as methanol,^[^ ^13^ ^]^ glycerol, and glucose. However, the intrinsic relationship between the oxidation state‐regulated M1‐O‐M2 structure in active species and the ^*^O reactive intermediate, as well as how to enhance HMF catalysis while inhibiting OER reactions, remains insufficiently studied.

Here, we constructed a V‐doped CoOOH layer on V‐doped CoP materials through an in situ activation method, aiming to promote the formation of V‐^*^O active intermediates, thereby achieving excellent HMFOR performance. The combination of experimental and theoretical calculations clarifies the key role of the V_H_‐O‐Co_H_ heterometallic oxygen‐bridge structure formed between the high‐valent V and low‐valent Co in V‐doped CoOOH layers in the generation of V‐^*^O, as well as its impact on HMFOR and OER. Meanwhile, the role of V‐^*^O in HMFOR catalysis was revealed. Based on this mechanism, the designed V‐doped Ni_2_P material effectively enhanced HMFOR performance, validating the feasibility of the proposed mechanism. Through the evaluation of efficient electrooxidation reactions of organic compounds such as glycerol, glucose, and methanol, we demonstrated the broad adaptability of the V_H_‐O‐Co_H_ structure in the conversion of various organic compounds.

## 2 Results

### 2.1 Synthesis and Characterization of V‐CoP/NF

**Figure 1:**
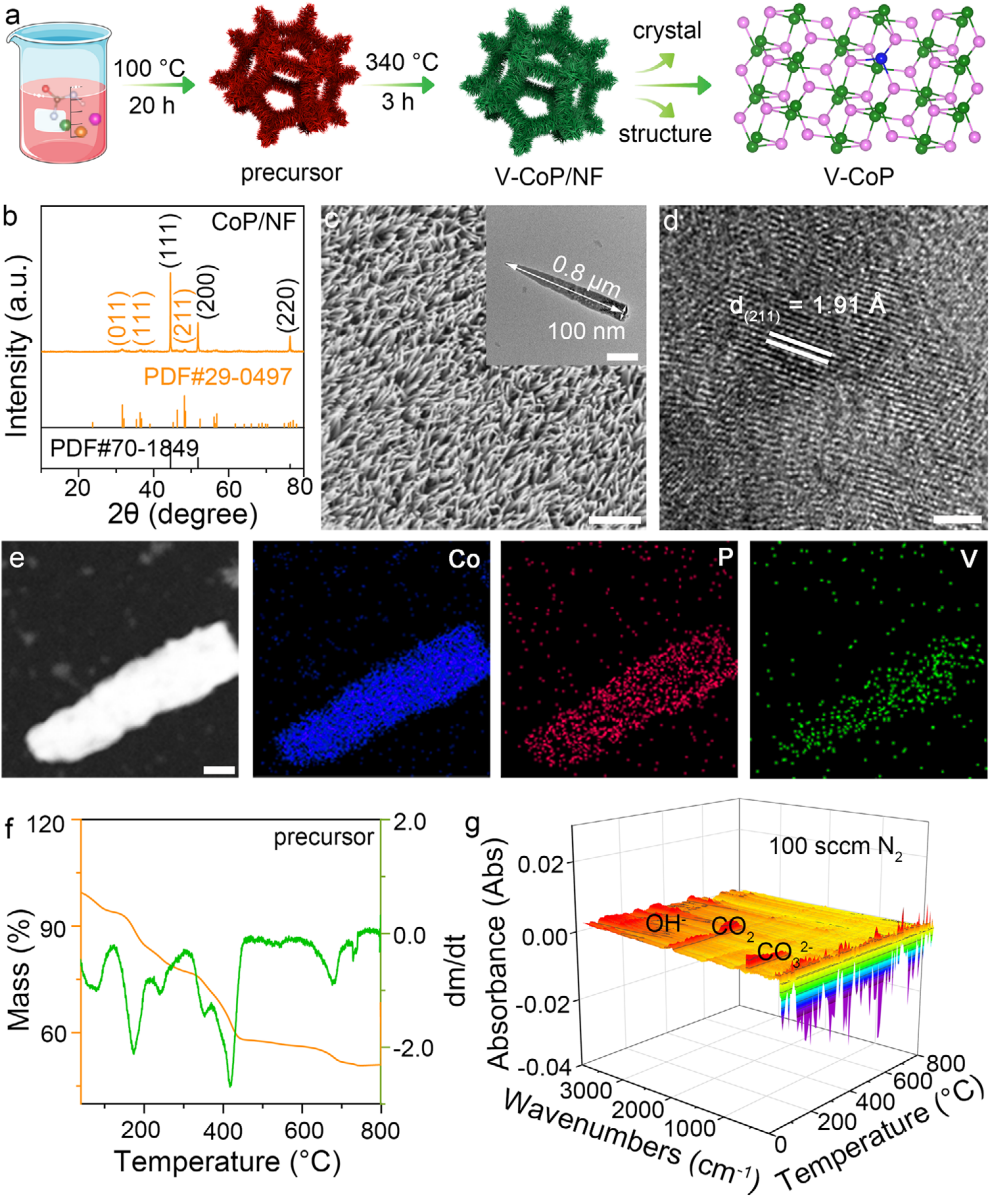
Synthesis schematic, morphology, and composition characterization of V‐CoP electrocatalyst. a) Schematic of the synthetic strategy of V‐CoP. b) XRD patterns of V‐CoP supported on Ni foam. c) SEM (scale bar: 2.0 µm) of V‐CoP/NF. Insert: Transmission electron microscopy (TEM) (scale bar: 200.0 nm) of V‐CoP. d) High‐resolution TEM (scale bar: 2.0 nm) and e) mapping image of V‐CoP (scale bar: 150.0 nm). Scraped precursor: f) Thermogravimetric analysis and g) thermogravimetric‐infrared (IR) spectrometry.

**Figure** **1**a shows that the V‐CoP catalyst is obtained by phosphatizing the V‐doped Co‐precursor treatment (Table S1 and Figure S1, Supporting Information). The detailed synthesis method is discussed in the experimental section. The X‐ray diffraction (XRD) data were analyzed to elucidate the crystal structure (Figure 1b). The three diffraction peaks at 44.5°, 51.8°, and 76.3° correspond to the typical peaks of nickel foam (NF, PDF#70‐1849). XRD indicated the crystal structure of the sample, and the main peaks corresponded to orthorhombic CoP (PDF#29‐0497). The doping of V induced a slight shift (negative shift of 0.03°) in the (011) peaks compared with the reference values of CoP. It is worth noting that the XPS data show that the valence state of Co is +2 and that of V is +4 (Figure S2, Supporting Information). According to previous reports, the V^4+^ radius was 0.58 Å, which was larger than that of Co^2+^ (0.55 Å),^[^ ^14^ ^]^ illustrating that the insertion of V^4+^ into the CoP lattice could cause lattice expansion. The successful doping of V was confirmed by transmission electron microscopy (TEM) and scanning transmission electron microscopy‐energy‐dispersive X‐ray spectroscopy (STEM‐EDS). The SEM and TEM images of V‐CoP (Figure 1c) show that nanowire arrays of diameters ≈100 nm are uniformly arranged on the nickel foam. The high‐resolution TEM (HRTEM) images show that V‐CoP exhibits clear lattice fringes with interplanar distances of 1.91 Å, which are 0.02 Å larger than the lattice spacing corresponding to the (211) crystal planes of CoP (Figure 1d). This result can be attributed to the lattice expansion of CoP caused by V doping, which is consistent with the XPS and XRD analyses. The STEM image and EDS map show that Co, P, and V are evenly distributed on the V‐CoP structure (Figure 1e). These results indicated that V was successfully doped into CoP.

We investigated the crystal growth mechanism using Thermogravimetric Analysis (TGA) and Fourier Transform Infrared Spectroscopy (FTIR), with a focus on the formation mechanism of V‐CoP during the thermal phosphating process. As shown in Figure S1 and Table S1 (Supporting Information), the successful incorporation of vanadium (V) under hydrothermal synthesis conditions is clearly observed. To further explore the impact of vanadium on the precursor decomposition and phosphating process, we conducted TGA‐FTIR analysis on both V‐doped cobalt precursor (V‐Co‐precurso) and cobalt precursor (Co‐precurso). Compared to Co‐precurso (Figure S3, Supporting Information), the proportion of CO_3_^2‐^ and OH^‐^ ions in V‐Co‐precurso is significantly increased, with a distinct two‐stage weight loss occurring before 400°C.^[^ ^15^ ^]^ This can be attributed to the decomposition of CO_3_^2‐^ and OH^‐^ ions. Sodium hypophosphite begins to decompose above 200°C, and the segmented decomposition process facilitates the continuous release of pH_3_ gas into V‐Co‐precurso, promoting the formation of V‐CoP (Figure 1f,g).

### 2.2 Electrochemical Performance Evaluation

In an H‐type cell with 1.0 m KOH aqueous solution (pH = 14) filled at the anode, as the HMF concentration increases, the current density significantly increases (Figure S4, Supporting Information), indicating that V‐CoP/NF exhibits good intrinsic catalytic activity for HMFOR. NF, V‐CoP/NF, and CoP/NF electrodes obtained cyclic voltammetry curves (5 cycles) in 100 mm HMF at a voltage range of 1.0‐1.5 V vs. RHE and a scan rate of 2 mV s^−1^ (Figure S5, Supporting Information). The test results indicate that the initial potential of NF within the test voltage range is 1.35 V, much higher than V‐CoP/NF (1.05 V) and CoP/NF (1.08 V). However, the maximum current density of NF is 13.1 mA∙cm^−2^, much lower than V‐CoP/NF (151 mA∙cm^−2^) and CoP/NF (67.6 mA∙cm^−2^). The above results indicate that nickel foam oxidation will not affect the LSV response of V‐CoP/NF.

**Figure 2:**
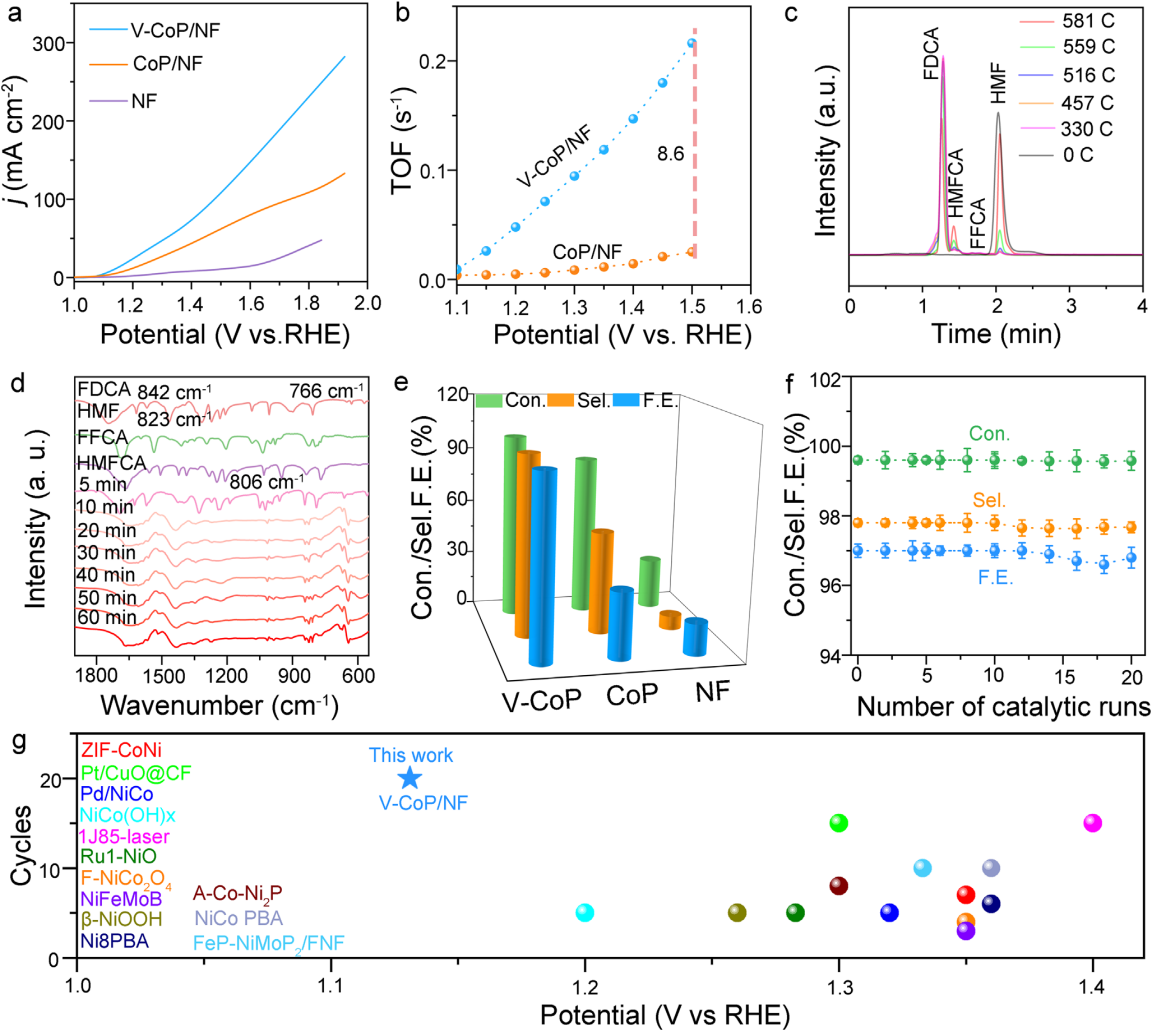
Electrochemical HMF performances in 0.1 m HMF+1 m KOH solution. a) LSV curves of a NF, CoP/NF, and V‐CoP/NF catalyst in 1 m KOH with 0.1 m HMF at a scanning rate of 5 mVs^−1^. b) TOF values of V‐CoP/NF and CoP/NF. c) The chromatogram of HMFOR. d) Infrared tracking spectra with different HMFOR time. e) HMF conversion, FDCA selectivity, and Faraday efficiency of V‐CoP, CoP, and NF. f) HMF conversion, FDCA selectivity, and Faraday efficiency of FDCA for V‐CoP/NF with 0.1 m HMF over 20 successive cycles at 1.30 V vs. RHE, 5.0 h for each cycle. g) The performances in comparison with the state‐of the art electrocatalysts for stability test at 10 mA cm^−2^. The error bar represents the standard deviation of four independent measurements (n=4, mean ± SD).

The electrochemical HMFOR performance of the prepared V‐CoP/NF, CoP/NF, and NF catalysts was examined with the addition of HMF (100.0 mm). When the HMF concentration is 100.0 mM, the required potential for V‐CoP/NF reaches 10.0 mA cm^−2^ at 1.13 V, which is significantly lower than that of CoP/NF (1.36 V) (**Figure** **2**a), and outperforms the value previously reported for the ZIF‐CoNi (1.35 V), Pt/CuO@CF (1.30 V), Pd/NiCo (1.32 V) and NiCo(OH)x (1.20 V) (Table S2, Supporting Information). Furthermore, the smaller the Tafel value, the faster is the increase in current density, leading to more favorable catalytic reaction kinetics.^[^ ^14^ ^]^ The Tafel slope of V‐CoP is 39.2 mV dec^−1^, which is superior to that of CoP/NF (83.9 mV dec^−1^, Figure S6, Supporting Information). This finding indicated that V‐CoP/NF was more favorable for accelerating the catalytic kinetics of HMFOR, highlighting its enhanced catalytic performance. To better investigate the intrinsic catalytic activity of V‐CoP, IR compensation LSV curves of V‐CoP with and without HMF were obtained in 1.0 m KOH (Figure S7, Supporting Information). The test results show that the overpotential of the IR compensation LSV curve for V‐CoP was lower than that of without IR compensation LSV curve with and without HMF, indicating that V‐CoP has better intrinsic catalytic activity. As shown in Figure 2b, the turnover frequency (TOF) of the catalyst exhibits a significant increase over the entire applied potential range (1.1–1.5 V). The V‐CoP/NF had a TOF value of 0.23 s^−1^ at 1.50 V, which was 8.6 times higher than that of CoP/NF (0.026 s^−1^). Therefore, V‐CoP/NF exhibited an excellent electrocatalytic HMFOR activity.

We selected industrially relevant concentrations of HMF (0.5, 0.7, 1 M) for the performance evaluation of V‐CoP/NF. The test results showed that the HMFOR activity of V‐CoP/NF gradually increased and the Tafel slope gradually decreased with increasing concentration, reflecting the intrinsic catalytic activity of the catalyst (Figure S8, Supporting Information). Here, we assume a first‐order reaction and investigate the relationship between the logarithm of concentration and reaction time in the HMF oxidation reaction. The experimental data show a linear relationship between the logarithm of industrially relevant concentration and reaction time (Figure S9, Supporting Information), indicating that this reaction is a first‐order reaction.

Next, the products of HMF oxidation were identified through liquid chromatography by analyzing compositional changes in HMF and its related oxidation products under varying current densities and reaction times. By adjusting the HMF concentration, only FDCA is detected as the target product, indicating that FDCA is the final catalytic product of HMF. Meanwhile, standard curves of HMF, HMFCA, FFCA, and FDCA were plotted in Figures S10–S13 (Supporting Information). The intermediate products formed during the catalytic process were investigated further by increasing the current density gradually. In Figure 2c, the HMFCA chromatographic peaks corresponding to the charge levels of 0, 516, 559, and 581 C are not visible. This is because before the reaction begins, only the reactant HMF (0 C) is present, and as the charge level increases, HMFCA gradually generates and is consumed (330C, 457C). When the charge reaches 559 and 581 C, HMFCA is almost completely depleted, so some peaks are invisible to HMFCA. For FFCA, the chromatographic peak intensity is very small at 330, 457, and 516 C, while the chromatographic peaks are not visible at 559 and 581 C. This is due to the reaction kinetics of FFCA conversion to FDCA is very fast. Therefore, the chromatographic peak intensity of FFCA is very small at low charges, and at high charges, FFCA has already been completely converted to FDCA, so the chromatographic peaks are not visible. The results showed that HMF was consumed gradually, leading to the formation of side products, such as HMFCA, FFCA, and FDCA. C_6_H_4_O_3_ (DFF) was not detected, supporting the hypothesis that FDCA was formed via the HMFCA pathway (Figures S14, S15, Supporting Information). When the charge reached 581 C, HMF was almost completely converted to FDCA (Figure 2c and Figure S16, Supporting Information).

In addition, the IR spectroscopy results showed the infrared peak corresponding to HMF at 823 cm^−1^ for 5–60 min (Figure 2d). The infrared peak corresponding to HMFCA at 842 cm^−1^ for 5‐60 min, and the intensity of the peak first increases and then decreases, confirming that HMF is quickly converted to HMFCA and gradually consumed. The infrared peak corresponding to FFCA at 766 cm^−1^ only appeared after 10 min of reaction, and the intensity of the peak was very low, confirming that FFCA was transformed from HMFCA after HMF oxidation for a period of time, and FFCA quickly converted to FDCA. The infrared peak corresponding to FDCA at 806 cm^−1^ only appeared after 30 min of reaction, and the intensity of the peak increased with time, indicating that HMF oxidation went through HMFCA to FFCA and then formed FDCA, and FDCA gradually accumulated. When the reaction time was extended to 60 min, the absorption peaks corresponding to FFCA and HMFCA gradually weakened and disappeared, whereas that of FDCA progressively intensified.^[^ ^16^ ^]^ In summary, HMF was converted to FDCA via the HMFCA pathway during catalysis.

HMF is converted to FDCA via the HMFCA pathway at a conversion rate (Con) of 99.6% and a Faradaic efficiency (Far) of 97.0% and a Selective efficiency (Sel) of 97.8% for V‐CoP/NF (Figure 2e), which significantly outperforms the CoP (Con: 86.6%, Far: 55.2%, and Sel: 35.9%) and NF catalyst. This finding indicated that V‐CoP/NF efficiently catalyzes the oxidation of HMF and controls the formation of side products, ensuring efficient yield for FDCA as the primary product. According to previous literature reports, ^[^ ^17^ ^]^ liquid chromatography‐mass spectrometry (LC‐Q‐MS) analysis showed signals at mass‐to‐charge ratios (m/z) 126, 142, and 156, which are attributed to the deprotonated molecular ions of HMFCA, FFCA, and FDCA, respectively. Quantitative analysis revealed that the products after 0.1 m HMF catalyzed oxidation were ≈97.4 mm FDCA, 0.3 mm FFCA, and 1.9 mm HMFCA (Figure S17, Supporting Information).

Besides excellent catalytic activity, the V‐CoP/NF catalyst also displayed outstanding catalytic durability. After twenty successive (one successive:1.38 V and 5.0 h, Figure S17, Supporting Information) HMFOR electrocatalytic cycles, the HMF conversion, FDCA yield, and Faradaic efficiency could be well maintained (Figure 2f). Electrolytes before and after the HMF catalytic oxidation reaction were tested using the activated V‐CoP/NF catalyst. The results showed that, compared to the initial state, no significant vanadium dissolution was observed in the electrolyte after 100 h of catalytic reaction (Table S3, Supporting Information). This indicates that the reconstructed sample possesses excellent stability, particularly in the stability of the Co_H_‐O‐V_H_ structure, providing assurance for long‐term, multiple, efficient, and stable HMF catalytic oxidation reactions.

In addition, we studied the relationship between V‐^*^O concentration and catalytic performance at different time points (2.0, 5.0, 20, 50, and 100 h) during the HMF catalytic process. The V‐^*^O concentration positively correlates with the intensity of in situ infrared measurements. Therefore, we set the V‐^*^O intensity at 1.38 V as a reference, assigning it a value of 1, and observed the changes in V‐^*^O intensity at other time points during the stability test (Figure S18, Supporting Information). The data show no significant change in V‐^*^O intensity, indicating that the V‐CoP/NF catalyst maintains its catalytic activity with no noticeable degradation during HMF catalytic oxidation, demonstrating outstanding catalytic performance. V‐CoP/NF also showed excellent FDCA yields and stability under similar conditions, compared to state‐of‐theart results reported previously (Figure 2g and Table S4, Supporting Information).

The electrochemical activity was evaluated of V‐CoP at different pH values (13, 11, 9), as well as the corresponding HMF conversion rate, FDCA selectivity, and Faraday efficiency. The test results show that the current densities corresponding to different pH values (13, 11, 9) at 1.42 V vs. RHE are 14.6, 0.68, and 0.42 mA· cm^−2^, respectively, which are all lower than the 85 mA· cm^−2^ corresponding to pH═14(Figure S19a–c, Supporting Information). Meanwhile, the HMF conversion rate, FDCA selectivity, and Faraday efficiency of V‐CoP at different pH values were (pH═13, Con.═59.9%, Sel.═49.7%, F.E.═38.1%), (pH═11, Con.═9.1%, Sel.═3.7%, F.E.═6.6%), and (pH═9, Con.═5.1%, Sel.═4.1%, F.E.═1.2%), respectively (Figure S19d, Supporting Information). The above results indicate that the electrochemical activity, HMF conversion rate, FDCA selectivity, and Faraday efficiency of V‐CoP electrode gradually decrease with the decrease of solution pH, suggesting that V‐CoP possess pH dependent during HMFOR process.

### 2.3 Formation of the V_H_‐O‐Co_H_ Structure

**Figure 3:**
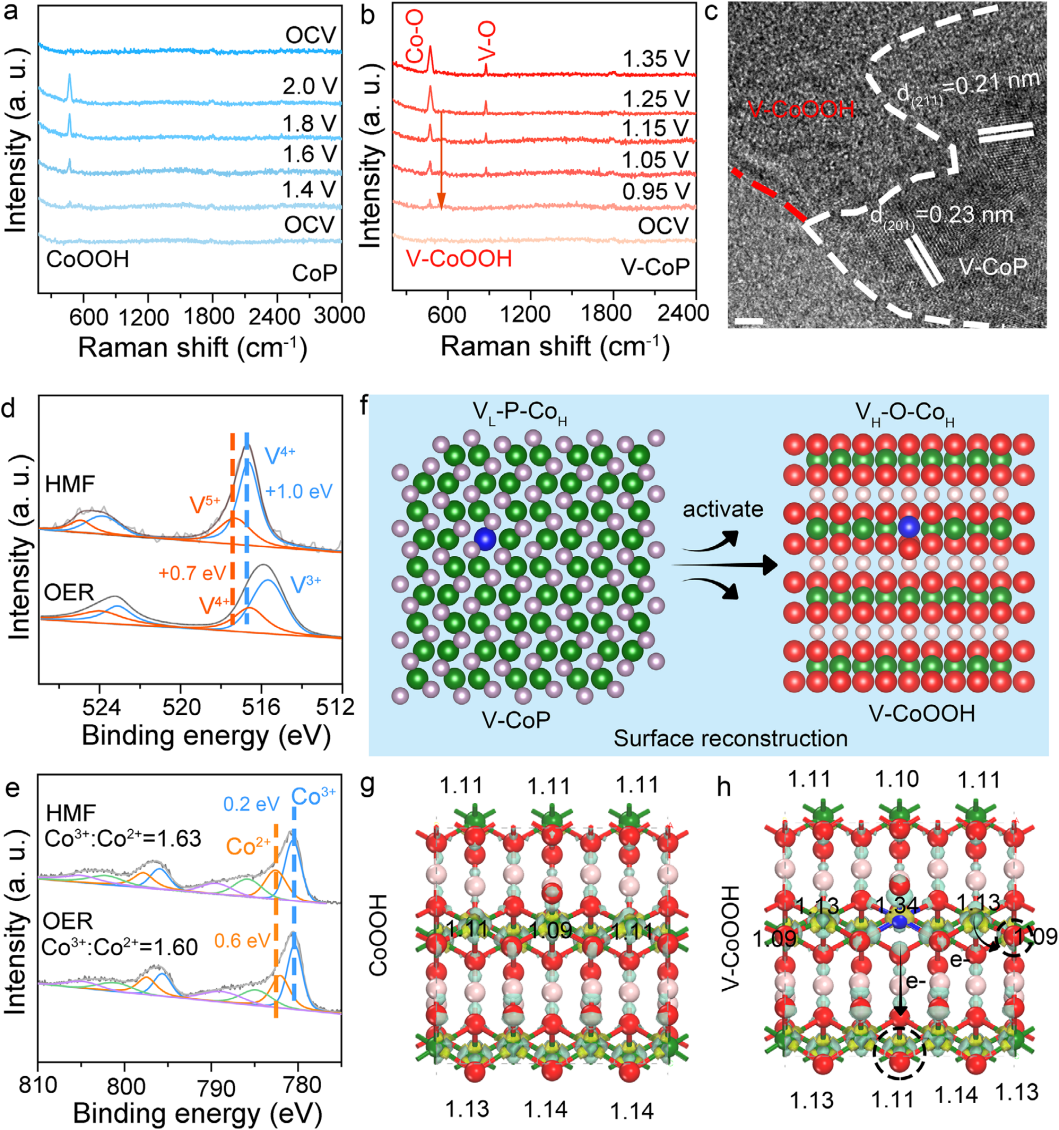
Experimental characterizations and Density Functional Theory for the formation of Co_H_‐O‐V_H_. In situ Raman spectroscopy of a) CoP/NF and b) V‐CoP/NF under elevating potentials with 0.1 m HMF+1.0 m KOH, respectively. c) HRTEM of V‐CoP after activation (scale bar: 2.0 nm). XPS survey of d) V and e) Co after the catalytic reactions of activated V‐CoP/NF in OER (electrolyte: 1.0 m KOH) and HMFOR (electrolyte: 1.0 m KOH + 0.1 m HMF). f) Schematic diagram of Co_H_‐O‐V_H_ formed by high‐oxidation state V and Co. g) Differential charge density of CoOOH catalyst (g) without and h) with V doping.

The changes on the catalyst surface of V‐CoP/NF during the HMFOR process were elucidated through characterization experiments. For comparison, the undoped catalyst was analyzed via in situ electrochemical Raman measurements to study the HMFOR process on the CoP surface (**Figure** **3**a). Raman spectra were recorded within a potential range of 0–2.0 V. No significant Raman peaks were observed in the potential range of 0–1.4 V vs. RHE. When the voltage increased to 1.6 V, a Raman peak near 474.5 cm^−1^ was observed, which corresponded to the stretching vibration peak of CoOOH.^[^ ^18^ ^]^ The intensity of this peak increased gradually at higher potentials. When the potential returned to the open‐circuit potential, the Raman peak near 474.5 cm^−1^ disappeared, indicating the instability of CoOOH and its dependence on the voltage.

We conducted in situ electrochemical surface Raman measurements on the V‐CoP/NF to observe the surface changes in the doped material during HMFOR (Figure 3b). Raman peaks were not detected at the open‐circuit voltage. As the potential increased to 0.95 V, a Raman peak was observed at 474.5 cm^−1^, which was attributed to the stretching vibration peak of the Co‐O bond (β‐CoOOH).^[^ ^19^ ^]^ High‐resolution TEM (HRTEM) images show the heterojunction region of V‐CoP and V‐CoOOH, indicating the presence of different phases on both sides of the interface. The lattice fringes have spacings of 2.31 and 1.91 Å, corresponding to the (201) and (211) planes of CoP, respectively. The amorphous region near CoP belongs to CoOOH, confirming that CoOOH and CoP are in contact to form a heterostructure catalyst (Figure 3c, Figure S20a, Supporting Information). This is consistent with the structural analysis from Raman spectroscopy. Subsequently, we tested the atomic force microscope (AFM) images of the catalyst, and the test results showed a weak heterojunction region between V‐CoP and V‐CoOOH (Figure S20b, Supporting Information). To better demonstrate the formation of heterojunctions, we tested Mott Schottky (MS) plots of V‐CoP and V‐CoOOH. The Mott Schottky (MS) diagram shows that pure V‐CoP (Figure S21a, Supporting Information) has a more negative flat band potential than V‐CoOOH (Figure S21b, Supporting Information), indicating that V‐CoP is a p‐type semiconductor and V‐CoOOH is an n‐type semiconductor. This result implies the electrons flow from n‐type V‐CoOOH to p‐type V‐CoP, when V‐CoP and V‐CoOOH are in intimate contact. As depicted in Figures S21c, S22 (Supporting Information), the heterojunction of V‐CoOOH and V‐CoP has a rectifying contact, with electrons flowing from the V‐CoOOH side with a lower Fermi level (EF) to the V‐CoP side, generating electron‐deficient V‐CoOOH due to the interfacial Schottky barrier.

At 1.05 V, a peak appears at 876.6 cm^‒1^, which is attributed to the V‐O bond.^[^ ^20^ ^]^ When V is undoped, CoP requires a potential of 1.6 V to collect the CoOOH characteristic signal. Notably, when V is doped, the CoOOH peak is detected at only 0.95 V, and as the voltage increases, the signal intensifies. This result suggests that V, as a dopant, facilitates the preferential reconstruction of Co. As the potential increased, the intensities of the stretching vibration peaks of the Co‐O and V‐O bonds increased significantly, confirming the formation of V‐O‐Co heterometallic oxygen‐bridge structure during the reconstruction process as a direct active species for HMFOR. EXAFS analysis performed on the V‐CoP sample post HMFOR reaction revealed distinct V‐O and Co‐O bonds at 1.52 and 1.66 Å, respectively. Compared with the Co‐P bond (1.65 Å) in CoP, these bonds confirm the formation of V‐O‐Co and suggest its role as a catalytic active species (Figure S23–S26 and Table S5, S6, Supporting Information).

To further investigate the V‐O‐Co structure, we conducted ex situ X‐ray photoelectron spectroscopy (XPS) analysis. XPS measurements were immediately performed on CoP/NF and V‐CoP/NF electrode materials after HMFOR and OER reactions. During HMF catalysis, V exhibited a higher oxidation state (V^5+^: 517.4 eV, V^4+^: 516.6 eV), while in the OER reaction without HMF, V was in a lower oxidation state (V^4+^: 516.6 eV, V^3+^: 515.7 eV), indicating that V participates in catalyzing the oxidation of HMF in its high oxidation state (Figure 3d).^[^ ^21^ ^]^ In contrast, Co (Co^3+^: 780.4 eV, Co^2+^: 782.1 eV) was found to be in a higher oxidation state during the HMFOR reaction, suggesting that Co participates in the HMF catalytic reaction in its higher oxidation state (Figure 3e).^[^ ^22^ ^]^ Therefore, the V‐O‐Co structure effectively catalyzes the oxidation of HMF in HMFOR through the high oxidation state of V and Co (Figure 3f).

To verify this, we selected CoOOH and V‐doped CoOOH as models (Figure S27, S28, Supporting Information) and performed Bader charge calculations to analyze the metal oxidation states. In the undoped CoOOH, the Bader charges of Co are +1.14 (high oxidation state) and +1.09 (low oxidation state) (Figure 3g). After doping with V, the Bader charge of V increases to +1.34, while the Bader charge of Co near V increases to +1.13 (Figure 3h). This change indicates that V doping significantly affects the charge distribution of Co. Regardless of whether high‐valent Co or low‐valent Co is replaced, V doping promotes the formation of the V_H_‐O‐Co_H_ structure (see Figures S27, S28 and Table S7, Supporting Information). This result is consistent with the XPS analysis and further validates the effectiveness of the V_H_‐O‐Co_H_ structure in hydrogen molecule‐forming (HMFOR) catalysis.^[^ ^22^ ^]^ Additionally, the differential charge density calculation was performed to visualize the direction of charge transfer. Yellow regions represent charge accumulation, while light green regions indicate charge depletion. The differential charge density map clearly shows the charge transfer from Co to the V‐O‐Co structure, further confirming the formation of high‐valent V‐O‐Co. Based on the above analysis, we conclude that V doping effectively tunes the electronic structure of Co and promotes the formation of the V_H_‐O‐Co_H_ structure, thereby enhancing catalytic performance.

### 2.4 Mechanistic Investigation

**Figure 4:**
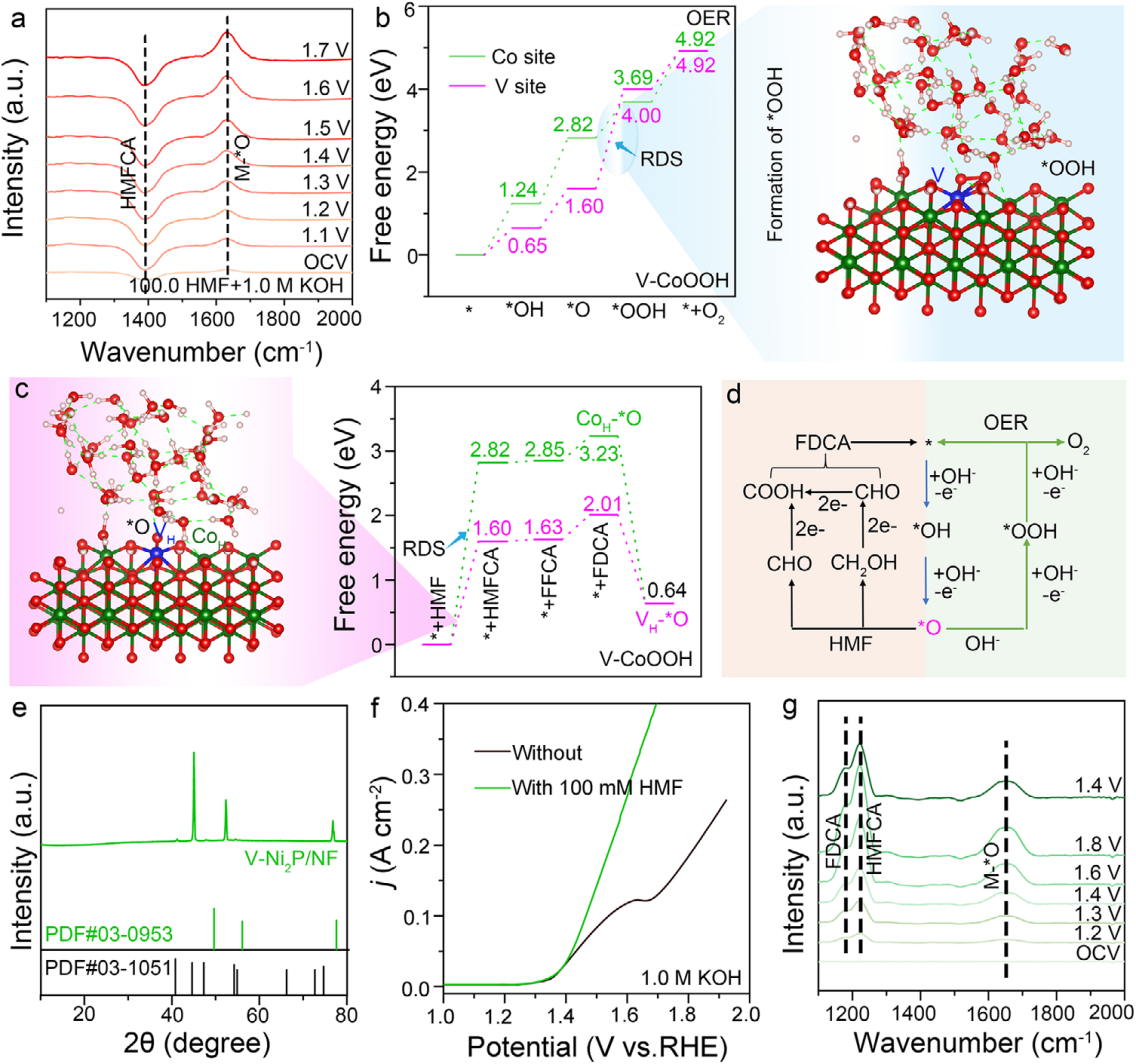
Experimental characteristics, DFT calculations, and universality of V‐^*^O formation. a) Electrochemical in situ IR spectra of HMFOR with V–CoP/NF in 0.1 m HMF+1.0 m KOH. b) Free energy diagram of OER intermediates at the V_H_ and Co_H_ sites in the CoOOH and V‐CoOOH model. Light blue: Theoretical model for the formation of the ^*^OOH intermediate corresponding to the rate‐determining step. c) Free energy diagrams for the catalytic conversion of HMF at the V_H_ and Co_H_ sites corresponding to M‐^*^O in the Co_H_‐O‐V_H_ model of V‐CoOOH. Pink: Theoretical model for V‐^*^O catalyzed HMF. d) Schematic diagram of the role of the V‐^*^O intermediate species formed in V_H_‐O‐Co_H_ in the catalytic oxidation of HMF and water. e) XRD patterns of V‐Ni_2_P supported on Ni foam. f) LSV curves of a V‐Ni_2_P/NF catalyst in 1 m KOH with and without 0.1 m HMF at a scanning rate of 5 mV s^−1^. g) Electrochemical in situ IR spectra of HMFOR with V‐Ni_2_P/NF in 0.1 m HMF+1.0 m KOH.

We investigated the role of reactive oxygen species adsorbed on the active sites in the catalytic HMFOR via in situ electrochemical IR spectroscopy measurements. According to previous literature, the peak at 1630 cm^−1^ corresponds to the characteristic peak of M‐^*^O. The peak corresponding to M‐^*^O is observed at 1630.0 cm^−1^,^[^ ^23^ ^]^ which gradually increases with the applied voltage, suggesting that ^*^O may also participate in HMF catalysis (**Figure** **4**a).

Additionally, to further verify the formation of M‐^*^O, we performed DEMS testing on the activated sample (V‐doped CoP) in 1.0 M KOH. Based on the provided DEMS data, we observed that the main component in the oxygen isotope signal is ^32^O_2_, while the signals for ^34^O_2_ and ^36^O_2_ are relatively weak. This phenomenon suggests that the oxygen generation primarily originates from ^16^O, with a lower involvement of ^18^O, implying that oxygen atom coupling does not play a significant role in the oxygen evolution process. This further supports the hypothesis that oxygen generation occurs via the AEM mechanism, with effective formation of the M‐^*^O intermediate, which is consistent with the infrared and DFT calculation data (Figure S29, Supporting Information).^[^ ^24^ ^]^ Therefore, these experimental data strongly support the speculation that the activated V‐doped CoP catalyst forms the V‐^*^O intermediate under the AEM mechanism.

In addition, we performed full infrared spectrum testing. According to previous literature, the absorption peak of M‐^*^OH typically appears in the 3000–3500 cm^−1^ range (Figures S30, S31, Supporting Information).^[^ ^11^ ^]^ From the full spectrum data, it can be observed that although the peak for M‐^*^OH is detected, its intensity is low and does not show significant changes with voltage variation. In contrast, the peak for M‐^*^O is more pronounced. Therefore, based on the overall analysis, the species with excellent catalytic activity is M‐^*^O.

^*^O species are the main active intermediates involved in the alkaline OER catalytic reaction. At the anode, there is a clear competition between the OER reaction and the HMF catalytic oxidation. During the HMF oxidation process using V‐CoP/NF, no gas evolution was detected (Figure S32, Supporting Information), and in situ electrochemical infrared analysis showed the formation of HMFCA intermediates (Figure 4a), indicating that HMF was effectively catalytically oxidized.^[^ ^17^ ^]^ To reveal the mechanism of V‐CoP/NF catalyzing HMF, we constructed CoOOH and V‐CoOOH models (Figure S28, Supporting Information), using Co and V as active sites, respectively, and considered the solvent effect to analyze the catalytic process involving intermediates such as ^*^OH, ^*^O, and ^*^OOH (Figure S33, Supporting Information). After V doping, the formation of ^*^OH and ^*^O was favored, but the formation of ^*^OOH was inhibited, leading to a decrease in the OER catalytic performance (Figure 4b and Figure S34, Supporting Information)^[^ ^25^ ^]^.

Through the detection of the M‐^*^O signal, we investigated the HMF catalytic oxidation process using V_H_‐^*^O and Co_H_‐^*^O as active catalytic sites (Figure 4c). The results show that V_H_‐^*^O significantly lowers the energy required for the HMF catalytic oxidation process (RDS: Co site: 2.81 eV, V site: 1.60 eV), thus improving catalytic efficiency and further proving the effectiveness of V_H_‐^*^O as an important intermediate species. To demonstrate the universality of the formation of the high‐valent Co‐O‐V structure by V substitution at the Co site, we selected the Co2 site, where V substitutes the high‐valent Co site (Figure S28, Supporting Information). The Bader charge values show that V has a charge of 1.35, while Co at positions 3 and 4 have Bader charge values of 1.15 (Table S7, Supporting Information), further confirming the formation of the high‐valent Co‐O‐V structure. We then selected V_H_‐^*^O (substituting Co2) as the active catalytic site to explore the HMF catalytic oxidation process. The results indicate that, compared to the Co1 site, there is no significant change in the energy required to overcome the HMF catalytic oxidation process (Figure S35, Supporting Information), again proving the effectiveness of the V_H_‐O‐Co_H_ structure formed during reconstruction as an important intermediate species.

In the undoped CoP, when Coh serves as the active site, the energy barrier required to overcome the rate‐determining step (RDS) is 2.82 eV, and the observed onset potential in the experiment is 1.36 V. However, when V_H_ serves as the active site, the energy barrier for the RDS decreases to 1.60 eV, and the corresponding experimental onset potential drops to 1.13 V. Considering the effects of solvation, there is a certain positive correlation between the energy barrier for the RDS and the overpotential. Based on this, DFT calculations can be used to preliminarily predict the performance of catalytic materials, providing theoretical support for the design of new HMF catalysts with low overpotentials.

We conducted a projected density of states (DOS) analysis to further investigate the impact of V_H_‐O‐Co_H_ on HMF oxidation (HMFOR) performance. The high‐valent V doping generates vacant d‐orbitals above the Fermi level, enabling the V_H_ atoms to more efficiently accept the lone pair electrons from OH‐. This finding enhances the adsorption and activation of OH‐ (Figure S36, Supporting Information). COHP analysis reveals that the O‐H bond on V_H_ (ICOHP ═ ‐7.82) is weaker than the O‐H bond on the Co_H_ site (ICOHP = ‐8.83), which facilitates the oxidation of ^*^OH to ^*^O (Figure S37, Supporting Information).^[^ ^26^ ^]^ This result further substantiates the effectiveness of V_H_‐^*^O in promoting the HMF catalytic mechanism. Based on these observations, we propose a reaction mechanism for HMF catalysis. Unlike the previously reported direct or indirect HMF catalytic mechanisms, we promote the formation of the V_H_‐^*^O intermediate by constructing a V_H_‐O‐Co_H_ bond. In this (Figures S38–S40, Supporting Information) structure, the high‐valent V acts as the active site, effectively adsorbing OH and accelerating the formation of ^*^O, which is the key active oxygen species in HMF catalysis. ^*^O interacts directly with HMF via the HMFCA pathway, generating intermediates HMFCA and FFCA, which are further catalyzed to effectively produce the final product, FDCA (Figure 4d).

To further validate the mechanism, we designed a method to introduce V into Ni_2_P, which was then treated with thermal phosphidation to obtain V‐Ni_2_P/NF (Figure 4e and Figure S41, Supporting Information). The activated samples were tested by LSV curves in 1.0 m KOH and 1.0 m KOH + 0.1 m HMF solutions at a slow scanning rate (5.0 mV s^−1^). The results showed that an overpotential of 1.31 V was required to achieve a current density of 10.0 mA cm^−2^ (Figure 4f). The excellent performance can be primarily attributed to the V‐O‐Ni structure, which promotes the formation of the V‐^*^O structure, consistent with the guidance of the proposed mechanism (Figure 4g). Notably, FDCA and HMFCA were effectively detected, confirming the successful catalytic oxidation of HMF. This phenomenon is similar to the one observed when V was doped into CoP, providing further experimental evidence for the validity of our mechanism.

We obtained the free energy of HMF catalytic transformation at the M‐^*^O sites in the Ni_H_‐O‐V_H_ model through DFT calculations. The results show that the free energy of V_H_ and Ni_H_ sites for HMF catalytic transformation in the Ni_H_‐O‐V_H_ model is greater than that in the Co_H_‐O‐V_H_ model. This suggests that the Co_H_‐O‐V_H_ model is more prone to oxidation reactions with HMF, meaning that V‐CoP in HMFOR requires a smaller overpotential than V‐Ni_2_P (Figure S42a, Supporting Information). Figure S42b–d (Supporting Information) shows that the double‐layer capacitance of V‐CoP is smaller than that of V‐Ni_2_P, suggesting that V‐Ni_2_P has a larger electrochemical active surface area, which benefits the kinetics of HMFOR.^[^ ^27^ ^]^ Therefore, V‐Ni_2_P exhibits a higher current density than V‐CoP in HMFOR.

We synthesized Mo/W‐doped CoP using the same method as the synthesis of V‐CoP, except that the vanadium salt was replaced with sodium molybdate (Figure S43, Supporting Information) and sodium tungstate (Figure S44, Supporting Information). Meanwhile, we compared the HMFOR activity of Mo/W‐doped CoP with V‐CoP.^[^ ^28^ ^]^ The test results indicate that compared with Mo/W‐doped CoP, V‐CoP has a smaller HMFOR oxidation overpotential at 100 mA cm^−2^ (Figure S45, Supporting Information). Subsequently, we tested the XPS of Mo/W‐doped CoP, and the results showed that there were high valence states of Mo/W (+4/+6) in the Mo and W 3d spectra, while Co 2p showed that the bottom valence state of Co^2+^ dominated (Figure S46, Supporting Information). This further indicates that only V‐CoP forms the V_H_‐O‐Co_H_ structure, while Mo/W‐doped CoP forms the Mo_H_‐O‐Co_L_ and W_H_‐O‐Co_L_ structures, demonstrating that the V‐^*^O intermediate is more stable and superior than Mo‐^*^O and W‐^*^O. Figure S46 (Supporting Information) shows that compared to Mo/W‐doped CoP, V‐CoP has a smaller Tafel slope. This is due to V^4+^/V^5+^ can form a V_H_‐O‐Co_H_ structure, while Mo/W‐doped both form low‐priced Co structures (Mo_H_‐O‐Co_L_ and W_H_‐O‐Co_L_ structures), resulting in better kinetic characteristics of V‐CoP.

### 2.5 Applied to Flow Electrochemical React

**Figure 5:**
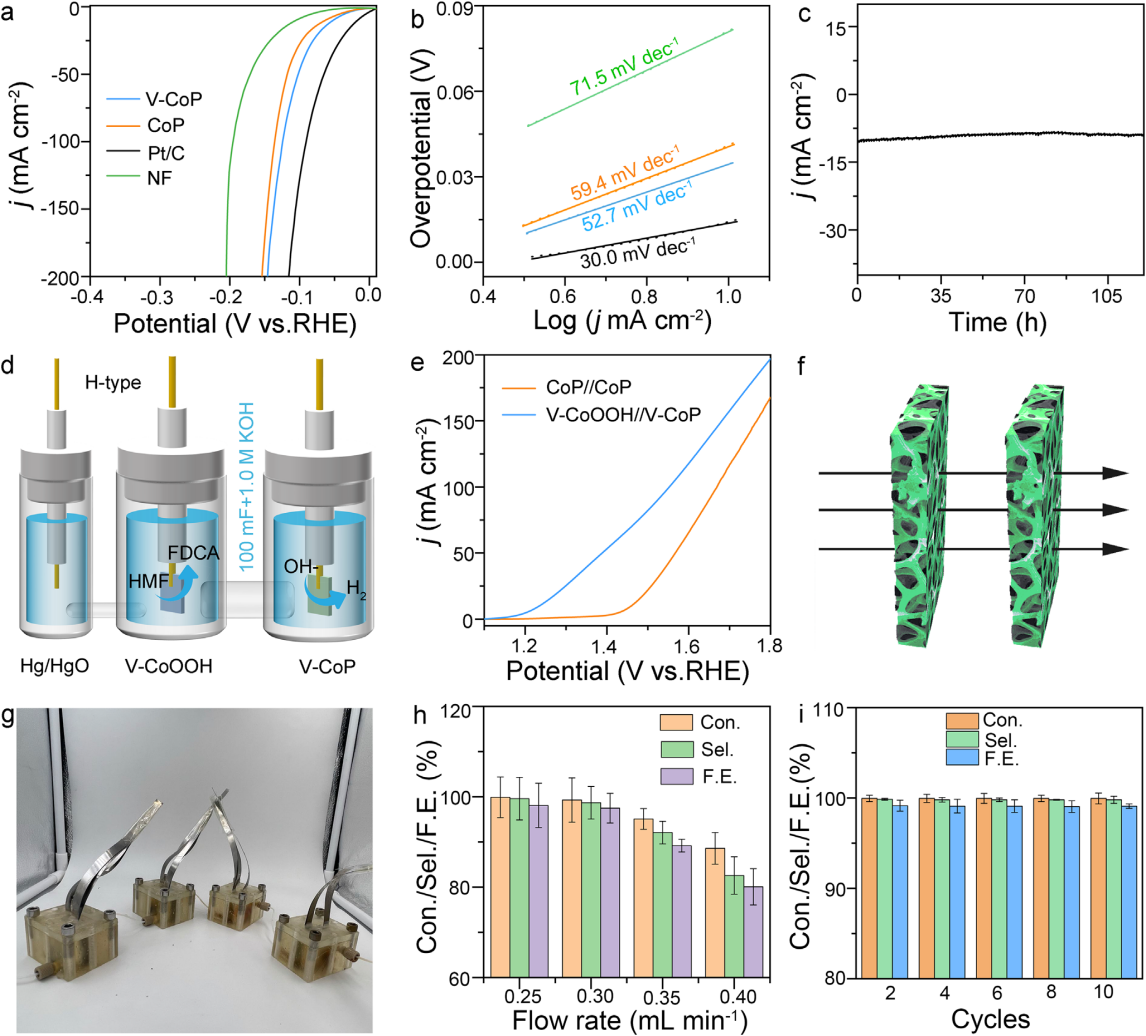
Electrochemical battery performance of V‐CoOOH//V‐CoP. Alkaline HER activity and cell performance of V‐CoP/NF. a) Polarization curves of HER for catalysts (V‐CoP/NF, CoP/NF, Pt/C, and NF). b) Tafel curves. c) Long‐term stability measurement of V‐CoP/NF for 120.0 h. d) Schematic of an electrocatalytic overall cell with V‐CoP/NF and restructured V‐CoP/NF as anode and cathode, respectively. e) Linear sweep voltammetry curves for the entire cell. f) Flow mode and g) physical model of flow‐through electrochemical reactor. h) HMFOR of flow‐through reactor with 0.3 mL· min^−1^ at 10 successive cycles. i) HMF conversion, FDCA selectivity, and FE at different flow rates in a flow‐through reactor. The error bar represents the standard deviation of four independent measurements (n=4, mean ± SD).

The cathodic reaction was crucial for the entire electrolytic cell. For the cathodic HER, the LSV curve of V‐CoP/NF was tested in 1.0 m KOH (pH═14). **Figure** **5**a shows that the V‐CoP/NF (‐41 mV with 10 mA cm^−2^) exhibits a slightly lower overpotential than Pt/C (‐19 mV with 10 mA cm^−2^), but superior to CoP/NF (‐65 mV with 10 mA cm^−2^) and NF (‐106 mV with 10 mA cm^−2^). Furthermore, V‐CoP/NF demonstrates a similar performance for the HER as compared with several recently reported catalysts (Figure S47, Supporting Information). The Tafel slope of V‐CoP/NF is 52.7 mV dec^−1^, which is superior to that of CoP/NF (59.4 mV dec^−1^) and the Tafel value under HER (Figure 5b), indicating a faster catalytic rate of V‐CoP/NF. However, V‐CoP/NF demonstrates a small decay over 110.0 h of continuous hydrogen production at 10 mA· cm^−2^ in alkaline solution (Figure 5c), indicating that V‐CoP/NF exhibits good hydrogen production stability. TEM (Figure S48a, Supporting Information) and HRTEM (Figure S48b) images show that no reconstruction layers are present on the V‐CoP/NF surface after HER, which further indicates that V‐CoP/NF has good structural stability during HER. In addition, a series of control experiments reveals the excellent HER activity of V‐CoP/NF (Figures S49–S50, Supporting Information).

Given their superior catalytic efficacy for the HMFOR and HER, they (V‐CoP/NF, CoP/NF) were used as the anode and cathode, respectively, in an H‐type electrolysis cell to evaluate the overall cell performance (Figure 5d). At a low scan rate of 5 mV s^−1^, the electrolyzer requires only 1.14 V to achieve a current density of 10.0 mA cm^−2^ (Figure 5e), outperforming the benchmark CoP/NF//CoP/NF catalyst and approaching the performance of the commercial Pt/C//V–CoP/NF catalyst (Figure S51, Supporting Information).

A continuous‐flow electrochemical reactor can effectively weaken the concentration polarization effect and increase the mass‐transfer efficiency of the reaction. In our previous research,^[^ ^17^, ^29^ ^]^ flow‐through reactors with porous electrodes reduced the thickness of the diffusion boundary layer to the radius of the pores or even smaller (1.0 µm) (Figure 5f,g). In this study, we fabricated a flow‐through reactor through computer design and 3D printing, and further conducted testing and evaluation of the HMFOR. We calculated that the volume of each unit reaction pool is ≈21mL (based on the fabrication of the flow‐through electrochemical reactor section). The oxidation retention times of the reaction solution in the flow reactor assembled with four units were 336, 280, 240, and 210 min at flow rates of 0.25, 0.3, 0.35, and 0.4 mL min^−1^, respectively. Therefore, when the retention time of the HMF electrolyte oxidation reaction is less than 280 min, the HMF oxidation activity will significantly decrease (Figure S52a, Supporting Information). We investigate the effect of electrode design on reaction efficiency by exploring the different contact flow rates between electrodes and reaction solutions (changing the angle between the electrode plane and the direction of electrolyte flow). The experimental results showed that when the angle between the electrolyte flow direction and the electrode plane was 90 °, the HMF conversion rate, FDCA selectivity, and Faraday efficiency reached 99.9%, 99.6%, and 98.1% (0.3 mL min^−1^), respectively. It is worth noting that as the angle gradually decreases, the HMF conversion rate, FDCA selectivity, and Faraday efficiency decrease accordingly (Figure S52b, Supporting Information).

In a flow‐through reactor comprising four reaction units, when the flow rate is less than or equal to 0.3 mL min^−1^, the conversion rate of HMF, selectivity for FDCA, and Faraday efficiency are above 90% (Figure 5h). When the flow rate was greater than 0.3 mL min^−1^, the HMF conversion rate, selectivity of FDCA, and Faraday efficiency exhibited varying degrees of attenuation, which may be attributed to the fast flow rate and insufficient oxidation retention time. Moreover, durability tests were conducted under conditions that included the use of a peristaltic pump in a flow‐through reactor with a flow rate of 0.3 mL min^−1^ (Figure S53, Supporting Information). After 10 cycles of stability assessment at 1.40 V, the conversion rate of HMF, selectivity of FDCA, and Faraday efficiency reduce by ≈1.0%, further underscoring the remarkable stability of this material system (Figure 5i).

To assess mass transfer limitations and scalability, HMFOR with industrial‐grade HMF concentrations was tested in a flow reactor. The experimental results showed that with the increase of HMF concentration (1, 1.5, 2 M), the conversion rate, selectivity, and Faraday efficiency all reached over 95%, which can be achieved with only a high flow rate (shorter retention time in the flow reactor), further confirming the feasibility of flow reaction for industrial production (Figure S54, Supporting Information).

Research on the Potential of V‐^*^O in Producing Different Types of Organic Industrial Raw Materials

**Figure 6:**
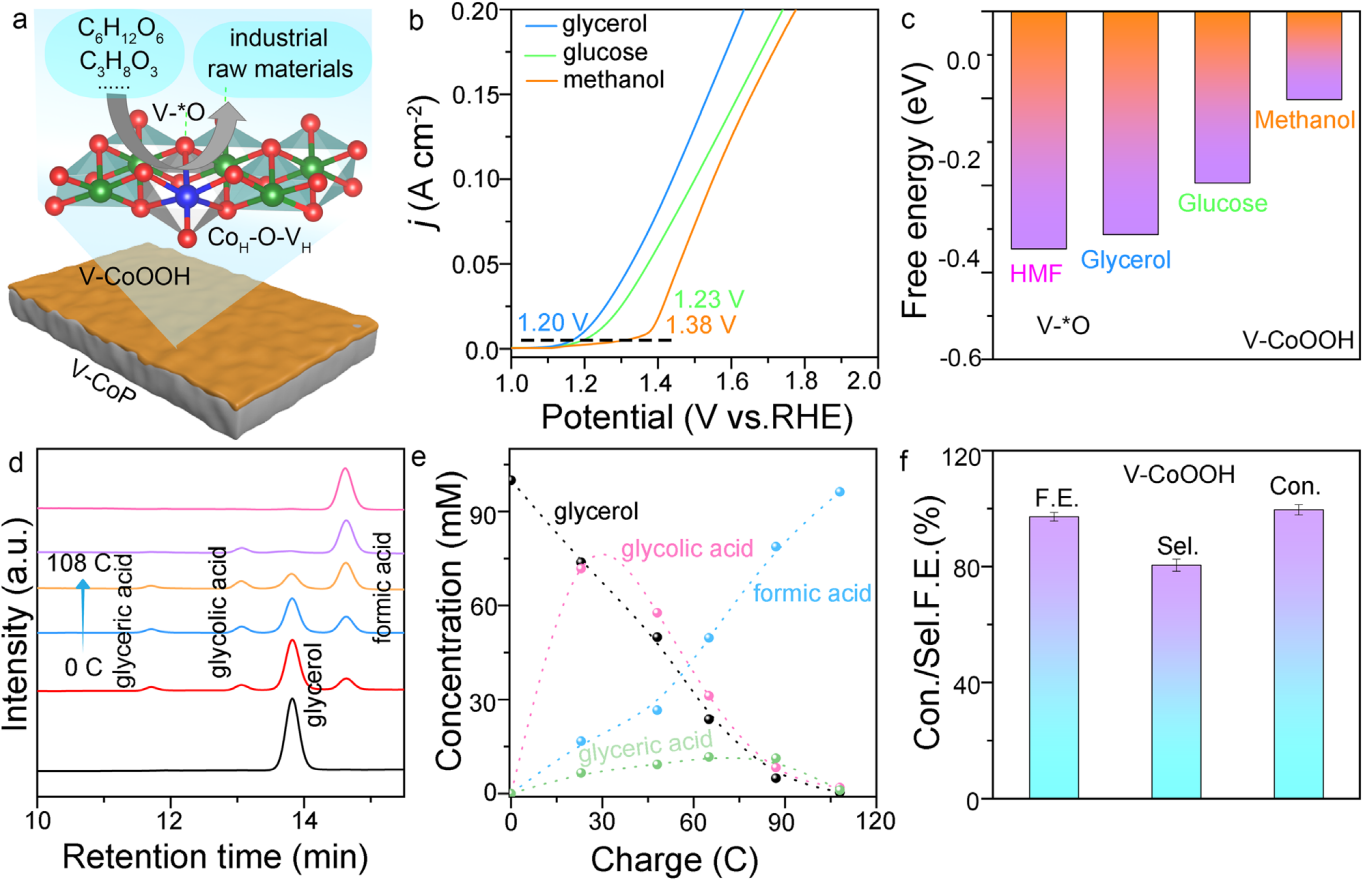
Exploration of the universality of V‐^*^O in the synthesis of multiple industrial feedstocks. a) Schematic diagram of the electrocatalytic oxidation of the active intermediate V‐^*^O, prepared by V_H_‐O‐Co_H_, to form multiple industrial feedstocks. b) LSV curves of a V‐CoP/NF catalyst in 1 m KOH with 0.1 m glycerol, glucose, and methanol at a scanning rate of 5 mV s^−1^. c) Comparison of the adsorption ability of the V‐^*^O intermediate on glycerol, glucose, and methanol. d) The chromatogram of the catalytic oxidation of glycerol. e) The variation curves of the concentrations of glycerol, glyceric acid, glycolic acid, and formic acid with increasing electricity. f) Glycerol conversion, formic acid selectivity, and Faraday efficiency of V‐CoP/NF. The error bar represents the standard deviation of four independent measurements (n=4, mean ± SD).

The V_H_‐O‐Co_H_ structure forms a V‐^*^O active intermediate that has a significant advantage in non‐direct adsorption catalysis. Theoretically, it can catalyze the oxidation of various organic compounds (such as glucose, glycerol, methanol, etc.), yielding different types of organic industrial raw materials (**Figure** **6**a).^[^ ^30^ ^]^ We used the V‐CoP/NF electrode to catalyze the oxidation of glycerol, glucose, and methanol, and observed that a current density of 10.0 mA cm^−2^ could be achieved with applied potentials of just 1.20, 1.23, and 1.38 V, demonstrating excellent electrocatalytic performance (Figure 6b). When catalyzing the oxidation of glucose, glycerol, and methanol using V‐^*^O as the active site, the performance differences are mainly attributed to the varying adsorption binding capabilities of V‐^*^O to these organic compounds (Figure 6c). Under a charge supply of 0‐125 C, the Faradaic efficiency for the conversion of methanol to formic acid reached 99.7%, with a conversion rate of 98.8% (Figure S55a,b, Supporting Information). Under 0‐603 C charge, the Faradaic efficiency of the conversion of glucose to glucaric acid reached 75.9%, and the conversion rate of 88.8% (Figure S55c,d, Supporting Information).

Based on this, we selected glycerol, which has the lowest overpotential, as the research subject. Under a charge supply of 0‐108 C, the Faradaic efficiency for the conversion of glycerol to formic acid reached 96.2%, with a conversion rate of 99.6% (Figure 6d–f). In conclusion, the V‐^*^O intermediate exhibited excellent catalytic performance, especially in the oxidation of glycerol, demonstrating high Faradaic efficiency and conversion rate. This proves its potential in the catalytic oxidation of organic compounds and provides a new approach for developing efficient and green organic oxidation methods, laying a foundation for related industrial applications.

## 3 Conclusion

We propose a novel catalytic strategy for the efficient conversion of HMF based on V‐^*^O, achieved through the manipulation of the oxidation state in the V_H_‐O‐Co_H_ structure, which successfully enables the effective formation of V‐^*^O and overcomes the limitations of traditional alkaline OER catalysis that hinder HMF conversion. By precisely tuning the heterometallic oxygen‐bridge structure of the oxidation state, the reconstructed V‐doped CoOOH surface significantly enhances the adsorption and activation of OH^‐^, promoting the oxidation of V‐^*^OH to V‐^*^O, thereby greatly improving the generation efficiency of V‐^*^O. This process facilitates the efficient binding of V‐^*^O with HMF and effectively suppresses the formation of ^*^OOH during OER catalysis, leading to a significant enhancement in the selectivity and catalytic performance of the V‐^*^O active intermediate toward HMF. Using this strategy, we achieved excellent catalytic performance for HMF, with an overpotential as low as 1.13 V (10.0 mA cm^−2^), and Faradaic efficiency, conversion rate, and selectivity close to 100%. The V‐Ni_2_P catalyst designed using this strategy further validates the authenticity of the V‐^*^O intermediate, demonstrating that V‐^*^O not only efficiently catalyzes the conversion of HMF but also effectively catalyzes the conversion of various organic compounds such as glycerol, glucose, and methanol. This showcases the excellent adaptability and immense potential of this catalytic system for the conversion of a wide range of organic compounds.

## Conflict of Interest

The authors declare no conflict of interest.

## Supporting information



Supporting Information

## Data Availability

The data that support the findings of this study are available in the supplementary material of this article.
