# Supplementary material for: High Oxidation State V‐O‐Co Structure Promotes the Role of V‐*O Intermediates in Efficient HMF Oxidation
Source: Adv Sci (Weinh). 2025 Aug 13;12(42):e08912. doi: 10.1002/advs.202508912 (PMC12622522; doi:10.1002/advs.202508912)
Supplement: Supplementary file 1 — Supporting Information [file ADVS-12-e08912-s001.docx]

**Supporting Information**

**High Oxidation State V-O-Co Structure Promotes the Role of V-*O Intermediates in Efficient HMF Oxidation**

Honglei Wang^* [a]^, Xue Wen^*[b]^, Jiuxiang Dai^[b]^, Dawei Xu ^[a]^, Xianzhu Luo^*[d]^, Pan Luo^*[c]^, Hongshuai Cao^*[b]^

Honglei Wang, Dawei Xu

College of Chemical Engineering and Technology, Key Laboratory of Catalytic Conversion Energy Coupling in Shanxi Province, Taiyuan University of Science and Technology, Taiyuan 030024, China.

Xue Wen^*^, Jiuxiang Dai, Hongshuai Cao^*^

School of Chemistry and Chemical Engineering; School of Electronics, Information and Electrical Engineering, Shanghai Jiao Tong University, Shanghai 200240, China.

E-mail: wenxue2020@sjtu.edu.cn; c207206@sjtu.edu.cn

Pan Luo^*^

Department of Plastic Surgery, Beijing Chaoyang Hospital Affiliated to Capital Medical University, Beijing, China.

E-mail: 15610691516@163.com

Xianzhu Luo*

Key Laboratory of Hainan Trauma and Disaster Rescue, Department of Wound Repair, The First Affiliated Hospital of Hainan Medical University, Hainan Medical University, Key Laboratory of Hainan Functional Materials and Molecular Imaging, College of Emergency and Trauma, Hainan Medical University, Haikou 571199, China.

E-mail: luoxianzhu@muhn.edu.cn

**Methods**

**Chemicals**

The nickel foam was purchased from Hebei Aegis Metal Materials Co., Ltd. (China). Cobalt nitrate (Co(NO_3_)_2_·6H_2_O), Nickel nitrate (Ni(NO_3_)_2_·6H_2_O), ammonium metavanadate (NH_4_VO_3_), ammonium fluoride, urea (CO(NH_2_)_2_), sodium monophosphate, chromatographic methanol, KOH, and furfural derivatives, including HMF, FDCA, FFCA, HMFCA, and DFF, were obtained from Sinopharm Chemical Regent Beijing Co., Ltd.

**Foam nickel preparation**

Initially, the foam nickel was cut into 1 cm × 1.5 cm samples, immersed in a solution containing 10 mL hydrochloric acid and 50 mL deionized water, and ultrasonicated for 30 min to remove the oxide layer on the surface. The samples were rinsed with deionized water until neutralized and then spin-dried. Subsequently, the solution was soaked in acetone/ethanol solution and sonicated for 10-30 min. Finally, it was rinsed with deionized water (currently in use) or allowed to dry naturally before immersion in anhydrous ethanol for later use.

**Electrodes preparation**

(1) Initially, 1.0 mmol cobalt salt, 3.0 mmol NH_4_F, 0.5 mmol NH_4_VO_3_, and 10.0 mmol CO(NH_2_)_2_ solutions were mixed with 0.035 L of water. Subsequently, the solution and Ni foam were placed in the reactor and maintained at 100.0 °C for 20.0 h. The V-doped Co precursor was then removed, washed several times with ethanol and water, and dried naturally. Next, the V-doped Co precursor was phosphated with 2.0 g NaH_2_PO_2_ in N_2_ at 340.0 °C for 3.0 h.

(2) The preparation of CoP was similar to that of V–CoP, except that ammonium metavanadate was not added during the hydrothermal step.

(3) Initially, 1.0 mmol nickel salt, 2.0 mmol NH_4_F, 0.5 mmol NH_4_VO_3_, and 15.0 mmol CO(NH_2_)_2_ solutions were mixed with 0.035 L of water. Subsequently, the solution and Ni foam were placed in the reactor and maintained at 95.0 °C for 10.0 h. The V-doped Co precursor was then removed, washed several times with ethanol and water, and dried naturally. Next, the V-doped Co precursor was phosphated with 1.0 g NaH_2_PO_2_ in N_2_ at 380.0 °C for 3.0 h.

**Characterization**

XRD technique was used to determine the crystal structures of the samples. TEM (JEM1011), and SEM (Hitachi S-4800) were used to explore the morphology and structure of the samples. XPS (PHI 5000 Verasa). The molecular vibrations or rotations of the samples were explored using Raman (SK100) and IR (IS50) spectroscopic techniques. The XAFS spectra of the V and Co K-edges were recorded at Shanghai Synchrotron Light Source.

**Electrochemical measurements**

All the electrochemical experiments were conducted on an electrochemical workstation (CHI 660E, Shanghai CH Instruments). All electrocatalysis experiments were measured in a flow cell with a three-electrode configuration, with as-synthesized catalysts as the working electrode and a reference electrode Hg/HgO (with 1 M KOH used as the filling solution) at the cathode and a piece of carbon as the counter-electrode at the anode. All the electrolytes were initially purged with Ar for 10 min, followed by the above tests. The tested potentials are calculated using the following formula:

RHE = E_Hg/HgO_ + 98 mV + 0.059 × pH. (Eq. 1)

where E represents the applied potential (V) relative to the corresponding reference electrode (RHE for a reversible hydrogen electrode and E_Hg/HgO_ for a Hg/HgO electrode). A certain amount of HMF (glycerol, glucose, and methanol) was dissolved in 1.0 M KOH, and linear sweep voltammetry (LSV) tests were conducted within the 0-1.0 V range. All the LSV curves were obtained at a scan rate of 2 mV s^-1^.

**Operational attenuated total reflection (ATR)-surface-enhanced IR absorption (SEIRA) spectroscopy measurements**

A Kel-F thin-layer spectroelectrochemical flow cell was used to study the O ad-species on the V–CoP/NF electrodes using SEIRA spectroscopy with an ATR configuration under well-defined flow conditions. The electrolysis composition consisted of 1.0 M KOH + 100.0 mF HMF. The V-CoP/NF (2.0 cm × 2.0 cm) electrode was used as the working electrode, which was pressed against a gasket of thickness approximately 200 µm and an inner diameter of 1.2 cm. Approximately 1.1 cm^2^ surface area of the V-CoP/NF electrode was exposed to the solution. The solution flowed into the top compartment through a central inlet capillary (diameter of 1 mm) and contacted the working electrode. Subsequently, the solution flowed out through six small capillaries (diameter of 0.5 mm) and an outlet capillary (diameter of 1 mm). The electrolyte flow rate was approximately 20 μL/s.

**In situ Raman**

In situ Raman spectroscopy of the sample surface and solution during the HMFOR process was performed using a confocal microscope Raman spectrometer and flow electrochemical reaction cell. The V-CoP/NF, platinum wire, and Hg/HgO electrodes were loaded into a flow electrochemical reaction cell containing an alkaline solution of 100 mM HMF. The flow rate of the electrolyte was maintained at 10 mL/min to effectively remove hydrogen gas that interfered with the test. Raman spectra were recorded after allowing the reaction to occur for 40 s at different constant potentials within the range 0–2.0 V. The obtained results were repeated three times under identical conditions to eliminate testing errors. Changes in the Raman spectra were observed at consistent potential intervals across the specified range.

**IR spectra test**

Real-time monitoring was performed by Fourier transform IR spectroscopy under constant potential conditions. The potential was set at 1.38 V relative to the RHE for the HMFOR for 60.0 min. Aliquots of the reaction solution (2.0 mL) were extracted every 5.0 min for spectral data analysis. The test was repeated three times under identical conditions to eliminate testing errors. Data were collected by subtracting the background peaks to minimize interference.

**Calculated details**

The first-principles calculations were performed using the Vienna Ab initio simulation package (VASP) based on the spin-polarized Density Functional Theory (DFT) (*Phys. Rev. B.* **1994**, *50*, 13181-13185). The projector augmented-wave (PAW) technique within the DFT framework was used (*Comput. Phys. Commun.* **2010**, *181*, 1862-1867), and the Perdew-Burke-Ernzerhof (PBE) functional were applied to deal with the exchange and correlation energy of all systems (*J. Phys. Chem.* **1996**, *25*, 1069-1111). Due to the surface reconstruction of the working electrode, newly generated hydroxyl oxides become true electrocatalysts. Therefore, we constructed a V-doped CoOOH model for theoretical calculations. The (10-10) crystal plane of CoOOH was exposed with one V atom replacing one Co atom on the surface. To avoid periodic mirror interference of reaction intermediates, the unit cell parameters are amplified to a=11.47 Å and b=12.94 Å, and a vacuum slab with a thickness of 15 Å was introduced. The plane-wave energy cutoff was set to 450 eV, and a (3×3×1) grid was applied for the sampling of Brillouin zone. We also considered the solvation effect based on the explicit solvation model by constructing a water molecule layer with a density of 1g/cm^3^ and a thickness of 8 Å. Geometry optimization process was repeated until the change of the total energy of two adjacent ionic steps is less than 10^-5^ eV and the force on the atoms less than -0.02 eV/Å.

The energy changes of HMF indirect oxidation and its potential competitive reaction that oxygen evolution reaction (OER) have been investigated. OER in alkaline solution proceeds via four successive reaction steps:

OH^−^ + * $\to$ *OH + e^−^  (R1)

OH^−^ + *OH $\to$ *O + H_2_O (l) + e^−^  (R2)

OH^−^ + *O $\to$ *OOH + e^−^ (R3)

OH^−^ + *OOH $\to$ * + O_2_ (g) + H_2_O (l) + e^−^  (R4)

The indirect oxidation of HMF via three reaction steps:

*O +HMF $\to$ *+HMFCA (R5)

*O +HMFCA $\to$ *+FFCA (R6)

*O +FFCA $\to$ *+FDCA (R7)

where * indicates an adsorption site; *OH, *O and *OOH are the oxygenated intermediates; O_2_ (g) and H_2_O (l) are gas-phase O_2_ and liquid-phase H_2_O, respectively. For each elementary step, the associated Gibbs free energy change (ΔG) was calculated to be Equation 1.

ΔG=ΔE+ΔE_ZPE-_TΔS-eU+0.0592pH (Eq. 2)

where ∆E is the electron energy difference obtained from the DFT calculation, ΔEZPE and ΔS are the change values of the zero-point energy and entropy, respectively, obtained from the vibration frequency calculation, and the entropy correction value of the gas molecule can be obtained from the standard database (*J. Electroana. Chem.* **2007**, *607*, 83–89). For each step containing an electron transfer from OH− to e^−^, corresponding free energy change can be calculated based on the standard hydrogen electrode (SHE) method developed by Nørskov et al. (*ACS Catal.* **2014**, *4*, 1148-1153):

OH^−^ − e^−^ $\to$ H_2_O − 1/2H_2_ (R8)

G(OH^−^) − G(e^−^) = G(H_2_O) − G(1/2H_2_) + kBTln10⋅pH (Eq. 3)

The last term is a correction to the Gibbs free energy of the OH^−^ anion at a certain pH value. Here, the temperature and pH are set to be 298.15 K and 14, respectively. kB is the Boltzmann constant.

**Electrochemical testing in flow-through reactors**

HMFOR tests were conducted in a flow reactor with three electrodes. The electrolyte flow rate was controlled at 0.3 mL/min. After ensuring smooth electrolyte flow and complete filling of the four reaction modules, LSV data were collected for testing. The tests were conducted thrice under identical conditions to ensure catalyst stability before data collection.

**HPLC tests**

HMF, HMFCA, FDCA, and FFCA were detected and analyzed using Agilent high-performance liquid chromatography with UV-visible detectors (wavelength of 265 nm) and C18 columns. In particular, 20.0 μL of reaction solution was neutralized with dilute hydrochloric acid to obtain 1 mL of resulting solution. Then, the resultant solution was poured in liquid chromatogram for detection. The ratio of ammonium formate to methanol in the mobile phase was 3:7 and the flow rate was 1mL/min. The analysis of glycerol oxidation products was obtained through Agilent chromatography and BioRad Aminex 87H column testing. Take 100 μL of electrolyte solution from the electrochemical cell, dilute it with dilute sulfuric acid solution to 1.0 mL (adjust the sample pH to 7.0), and then inject 10 μL of diluted sample directly into the BioRad Aminex 87H column. In equal gradient mode, use 5 mM H_2_SO_4_ solution as the mobile phase and set the flow rate to 0.5 mL min^-1^. The conversion, selectivity, and Faradaic efficiency are calculated using the following formulas:

$$\text{HMF conversion (\%) }\frac{\text{mol (HMF transformed)}}{\text{mol (initial HMF)}}\text{ 100 \% (Eq. 4)}$$

$$\text{FDCA selectivity}\text{ (\%) }\frac{\text{mol (FDCA synthetic)}}{\text{mol (HMF transformed)}}\text{ 100 \% (Eq. 5)}$$

$$\text{Faradaic efficiency}\text{ (\%) }\frac{\text{mol (FDCA synthetic) }\text{ 6F}}{\text{total charge}}\text{ 100 \% (Eq. 6)}$$

$$\text{Glycerol conversion (\%) }\frac{\text{mol (Glycerol transformed)}}{\text{mol (initial Glycerol)}}\text{ 100 \% (Eq. 7)}$$

$$\text{Formic acid selectivity}\text{ (\%) }\frac{\text{mol (Formic acid synthetic)}}{\text{mol (Glycerol transformed)}}\text{ 100 \% (Eq. 8)}$$

$$\text{Faradaic efficiency}\text{ (\%) }\frac{\text{ (n}\text{Glyceric}\text{4+n}\text{Glycolic}\left( \frac{\text{2}}{\text{3}} \right)\text{5}+\text{n}\text{Formic}\left( \frac{\text{1}}{\text{3}} \right)\text{8) }\text{ 6F}}{\text{total charge}}\text{ }$$

$$\text{100 \% (Eq. 9)}$$

Where F is 96485 C mol^-1^, n_glyceric_, n_glycolic_, and n_formic_ are the moles (mol) of respective glycerol oxidation reaction products.

TOF **tests**

The intrinsic catalytic activity is measured by the turnover frequency (TOF) for each active site. We attempted to quantify the active sites by electrochemistry. Firstly, cyclic voltammograms of V-CoP, CoP, and Ni catalysts were obtained in the range of 0.2 V to 0.6 V vs. RHE at pH=7.0. Secondly, utilize the integrated charge over the whole potential range should be proportional to the total number of active sites as the basis for evaluating active sites. Assuming that both reduction and oxidation are single electron processes, the upper limit of active sites on the surface of each catalyst can be calculated. Finally, the polarization curve normalized by the active site under pH=14.0 was obtained and represented as TOF. Meanwhile, comparing the TOF values at the same different potential is used to evaluate the number of active sites for each catalyst.”

The “The turnover frequencies (s^-1^) were calculated follow:

TOF= $\frac{\text{I}}{\text{6}\text{Fn}}$

where I is current (A) during polarization curve measurement. F is Faraday constant (C/mol). n is number of active sites (mol). 1/6 is based on the fact that forming a FDCA molecule requires six electrons.

**Process of transferring XPS samples**

a. Inert Atmosphere Protected Transfer: After the sample is removed from the electrolytic cell, it is immediately placed in an inert gas atmosphere, typically using nitrogen or argon. To prevent the sample from being exposed to air, an atmosphere-protective container is used to ensure that the sample does not come into contact with air during the entire transfer process, thus avoiding oxidation or moisture contamination.

b. Sealed Transfer Container: The sample is carefully placed into a sealed transfer container, which is filled with inert gas. The transfer container undergoes an airtightness check to ensure that the atmosphere remains stable during the transfer process.

c. Vacuum Transfer System: To further reduce the chances of air contact during the transfer process, the sample is transferred to the XPS instrument through a vacuum transfer system. This system moves the sample from the protective atmosphere to the XPS analysis chamber, maintaining a low-oxygen environment throughout the process to ensure that the sample surface remains uncontaminated.

d. Sample Installation: The sample is precisely installed on the XPS sample stage, ensuring that the sample surface is accurately aligned with the XPS beam, avoiding any deviation. This process is conducted entirely under an inert atmosphere to prevent the sample from coming into contact with air, thereby ensuring the reliability of the XPS analysis data.

**Statistical Analysis**

Data Pre-processing: Prior to data analysis, the data were pre-processed to ensure accuracy and consistency. Outliers were identified using boxplots, and obvious outliers were removed. Data normalization was performed using z-score normalization to ensure comparability across different measurement conditions. Data Presentation: The data are presented as mean ± standard deviation (SD), which reflects the results of each experiment along with the degree of dispersion. Sample Size (n): The sample size for each electrocatalytic performance measurement was [specific value]. All data are based on independent measurements with a repetition of more than 3 times. Statistical Methods: Statistical significance was assessed using ANOVA or other appropriate tests. Before testing, the normality and homogeneity of variance assumptions were confirmed to be valid.


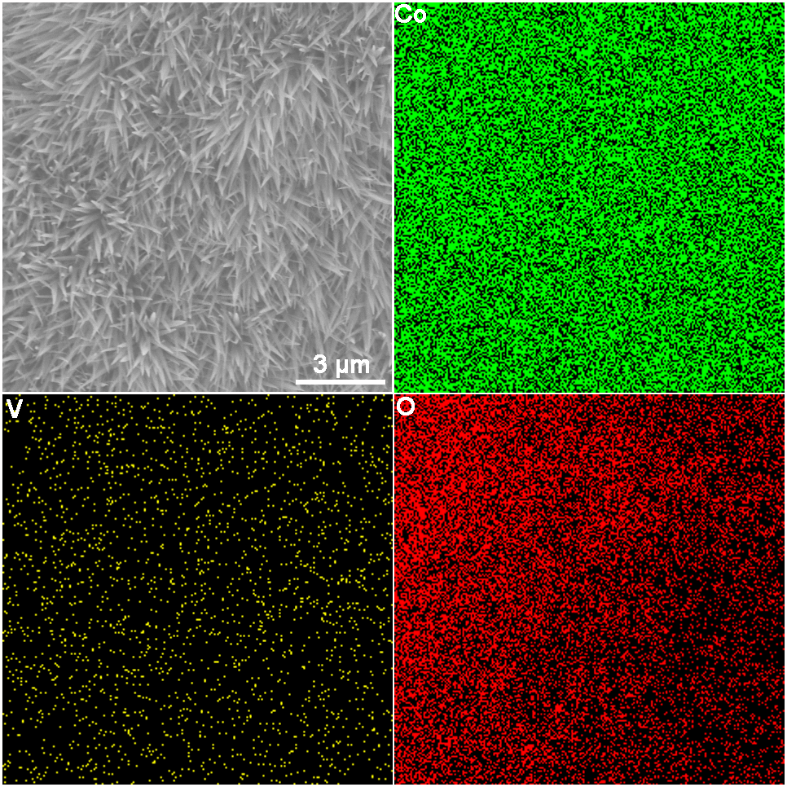


**Figure S1** The SEM images elements mapping of V-doped Co-precursor.

**Table S1** The mass percentage and atomic percentage of elements in V-doped Co precursor.

| Elements | Mass ratio | Atomic |
| --- | --- | --- |
| Co | 68.98 | 37.79 |
| V | 0.29 | 0.18 |
| O | 30.73 | 62.03 |
| Total | 100 | 100 |


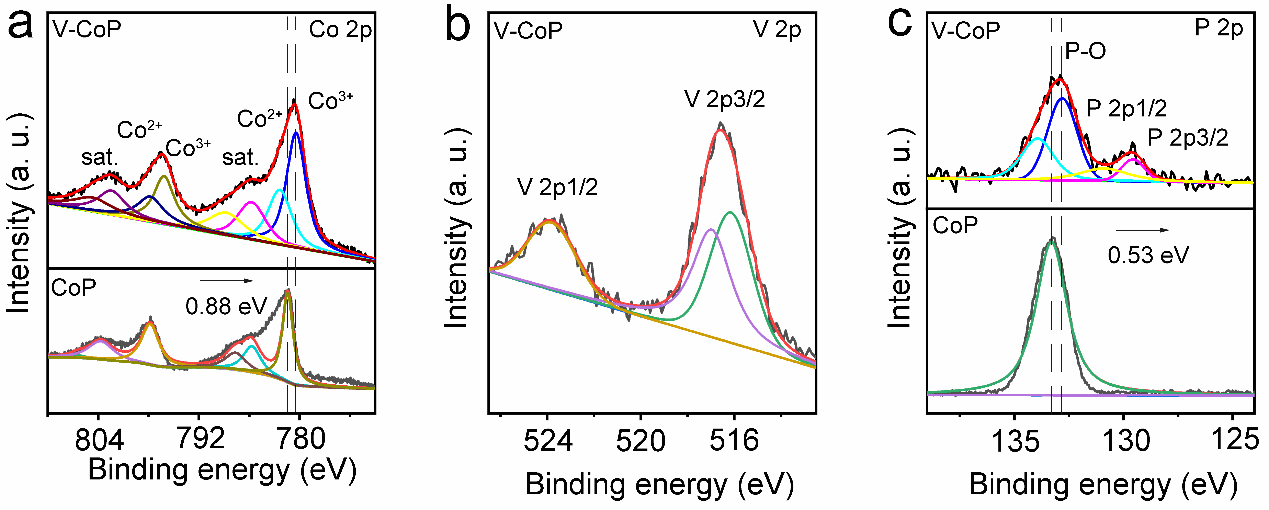


**Figure S2** XPS spectra of (a) Co 2p, (b) V 2p, and (c) P 2p of V-CoP and CoP.

*
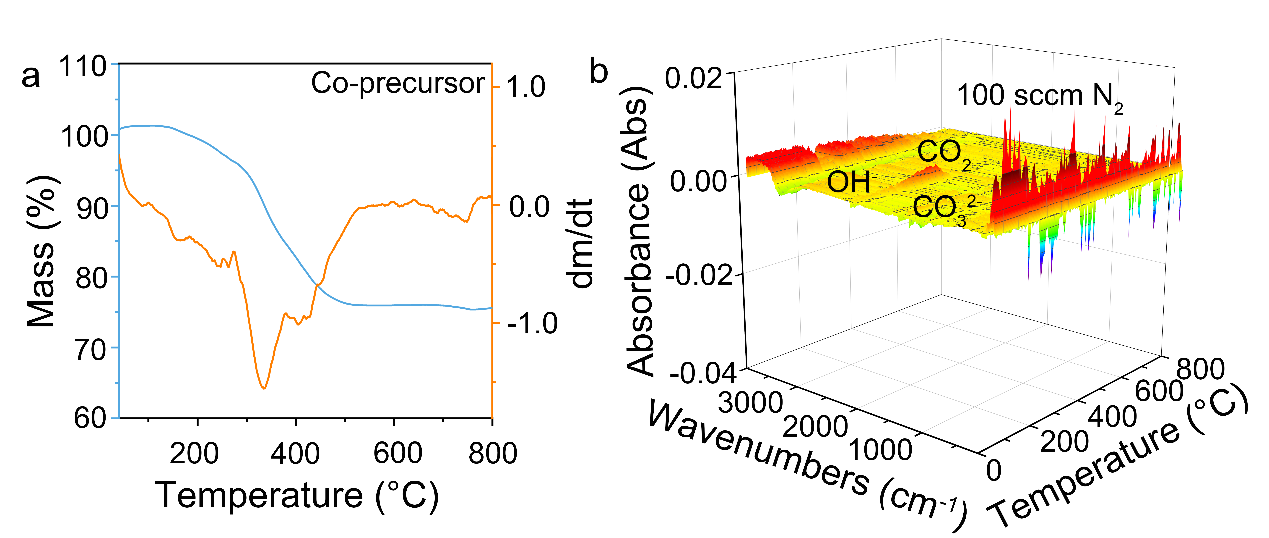
*

**Figure S3** Scraped Co-precursor: (a) Thermogravimetric analysis and (b) thermogravimetric-infrared (IR) spectrometry.


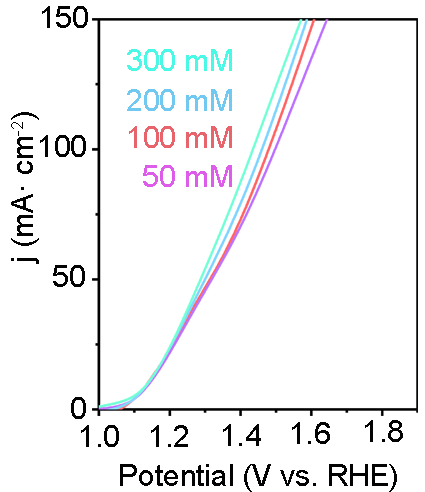


**Figure S4** Polarization curves for different concentrations of V-CoP for HMFOR.


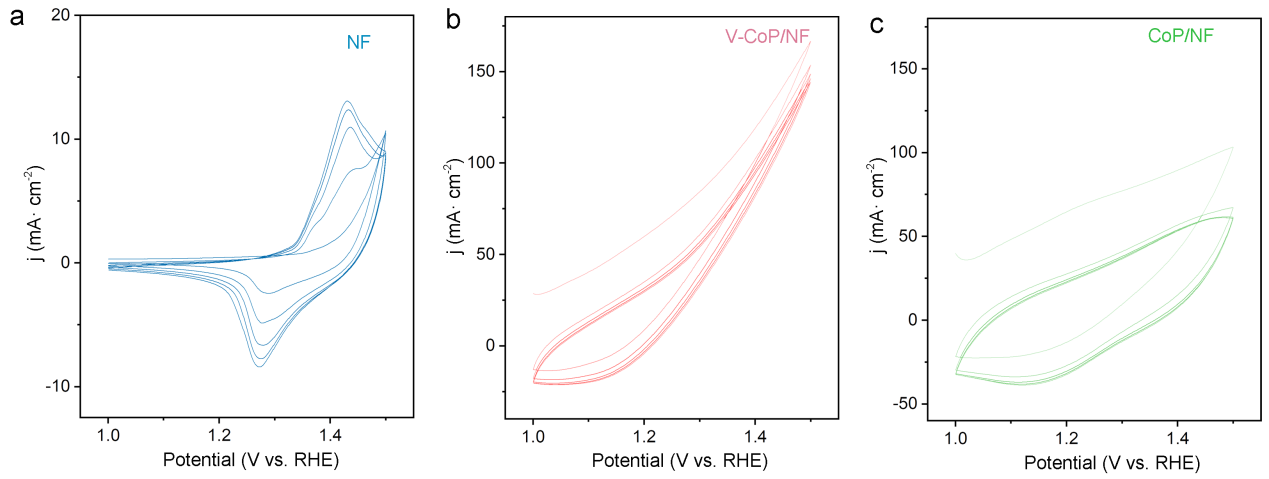


**Figure S5** The cyclic voltammograms of (a) NF, (b) V-CoP/NF, and (c) CoP/NF in the range of 1.0-1.5 V vs. RHE (5 cycles).

The results show that no Ni oxidation peak appears in the 1 M KOH solution containing 0.1 M HMF. The corresponding data has been added to Figure 2a. However, the LSV curve of CoP/NF in a solution containing only 1 M KOH still shows the Ni oxidation peak. This is because the oxidation potential of Ni is higher than that of HMFOR, so the Ni oxidation peak is not observed in the LSV curve of CoP/NF in HMFOR (Figure S5c and Figure 2a).


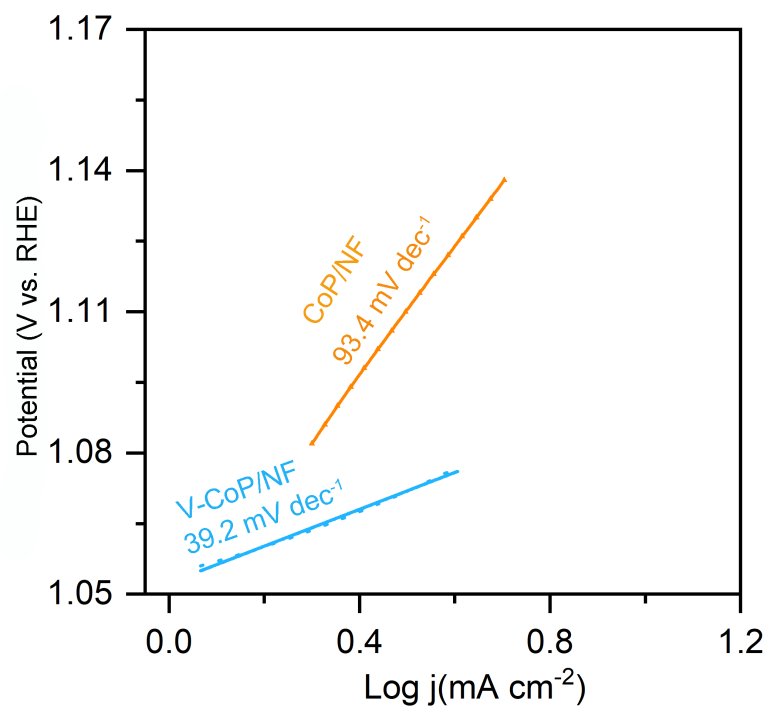


**Figure S6** Tafel slopes for HMFOR.


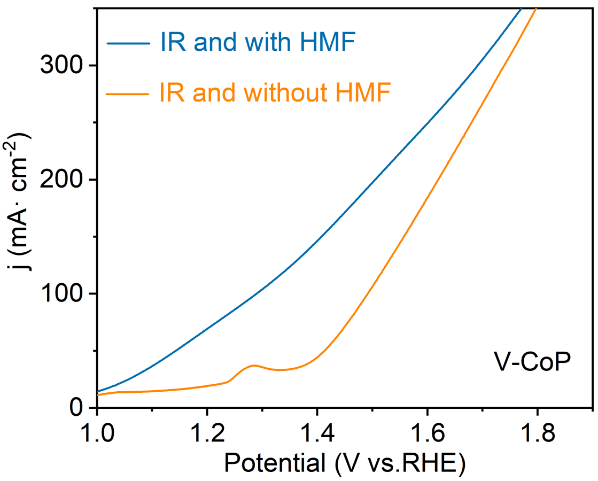


**Figure S7** The IR compensation LSV curves of V-CoP with and without HMF.

In Figure S7, the LSV curve is after IR compensation, whereas in Figure 2a, the LSV curve is without IR compensation. As a result, the LSV curve in Figure S7 shows a current density of 0.3 A·cm⁻² at 1.7 V vs. RHE, while the LSV curve in Figure 2a does not reach a current density of 0.3 A·cm⁻² even at 1.9 V vs. RHE.


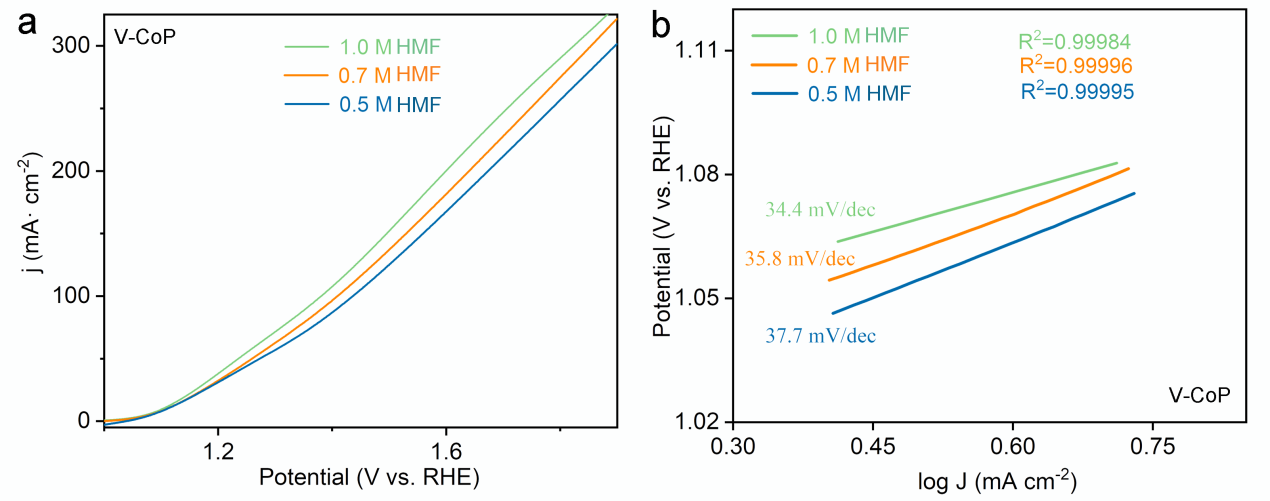


**Figure S8** (a) HMOFR polarization curve of V-CoP at industrial concentration level (HMF ≥ 0.5 M). (b) The Tafel curve of V-CoP corresponding to industrial concentration levels.

*
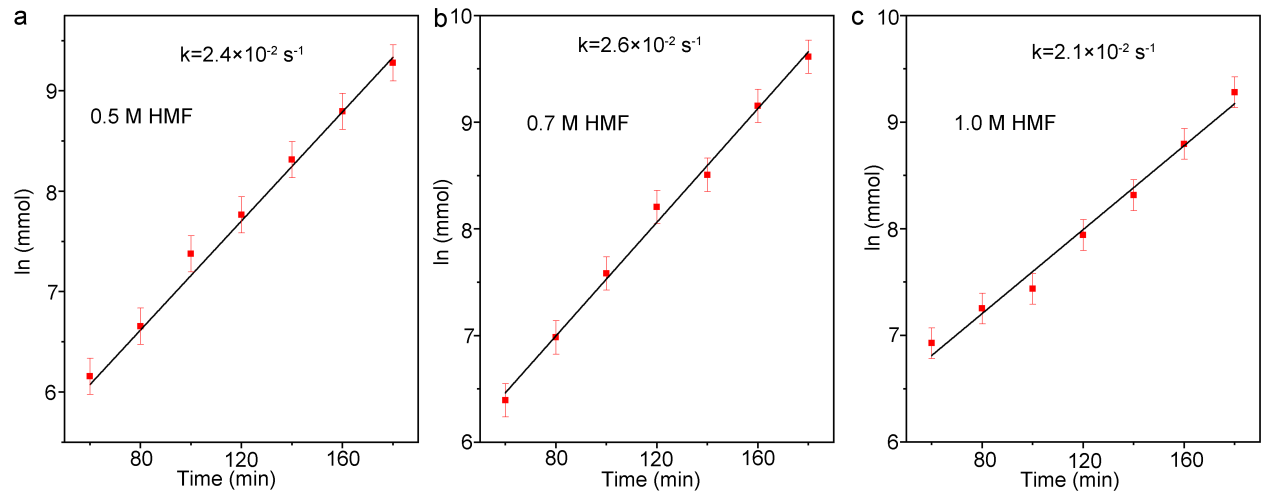
*

**Figure S9** Relationship between logarithmic concentration and time variation at HMF concentrations of (a) 0.5 M, (b) 0.7 M, and (c) 1.0 M. The error bar represents the standard deviation of four independent measurements (n=4, mean ± SD).

*
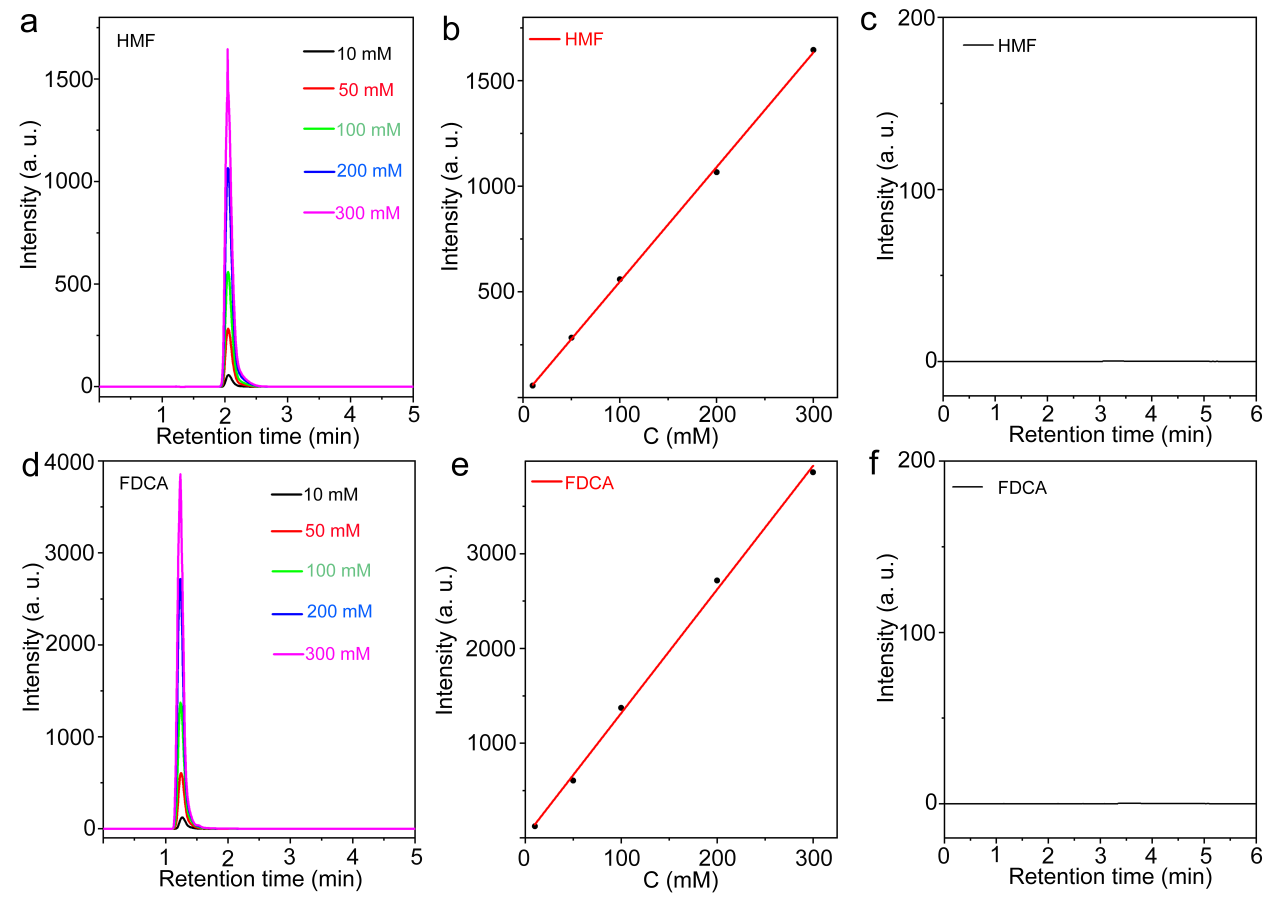
*

**Figure S10** Calibration curves and detection limits of the HPLC for HMF and FDCA. (a) Liquid phase detection intensity of HMF at different concentrations. (b) The linear relationship between the concentration of HMF and its liquid-phase detection intensity. (c) The liquid phase detection intensity curve corresponding to the HMF concentration at the intersection of the reverse extension line of the straight line in Figure b and the x-axis. (d) Liquid phase detection intensity of FDCA at different concentrations. (e) The linear relationship between the concentration of FDCA and its liquid-phase detection intensity. (f) The liquid phase detection intensity curve corresponding to the FDCA concentration at the intersection of the reverse extension line of the straight line in Figure e and the x-axis.

Figure S10 shows the liquid-phase detection curve at the concentration corresponding to the intersection of the inverse extrapolation line of the FDCA concentration vs. liquid-phase detection intensity and the x-axis. The results indicate that the liquid-phase detection intensity at this concentration approaches zero, thus confirming that this concentration represents the detection limit of FDCA in liquid chromatography. In summary, Figures S10(c) and (f) show liquid-phase tests at this concentration.

*
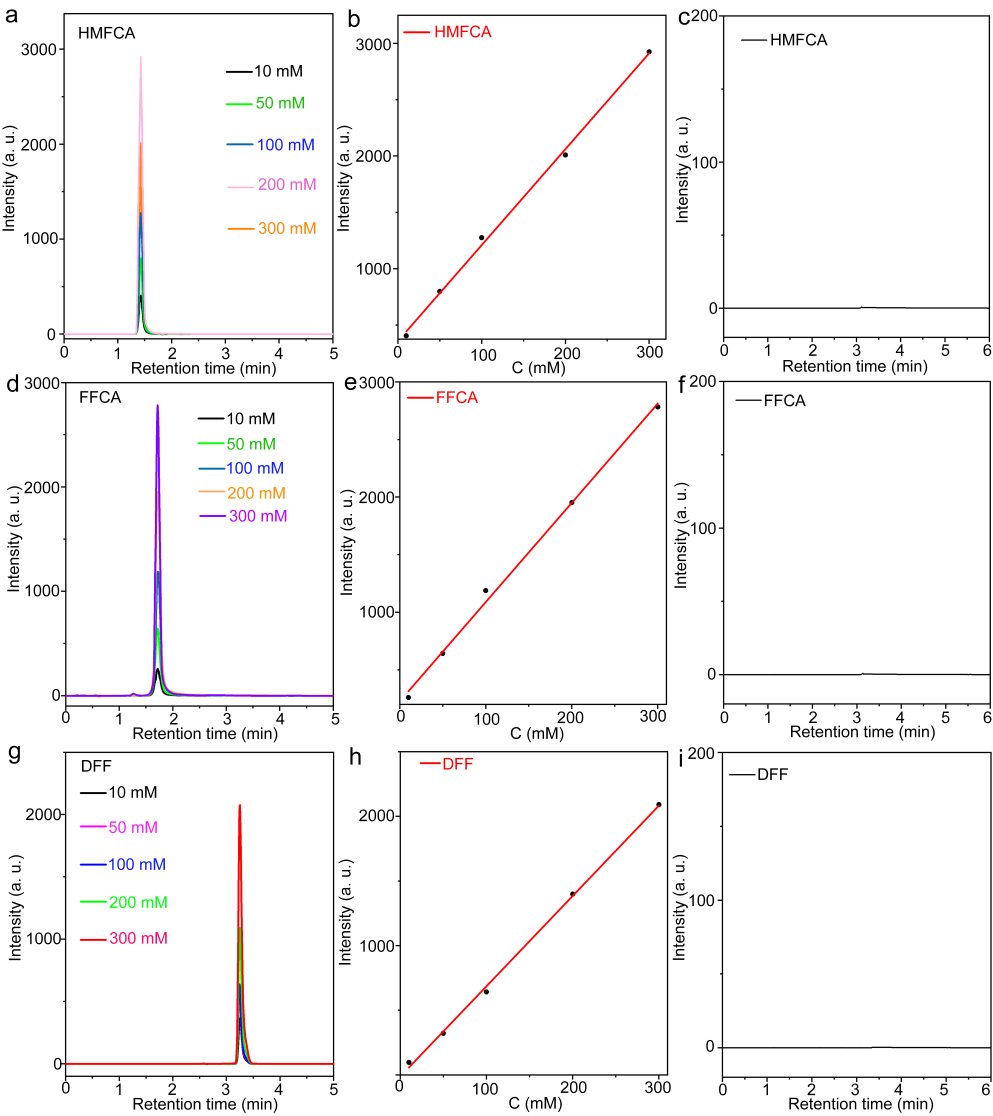
*

**Figure S11** Calibration curves and detection limits of intermediates HMFCA, FFCA, and DFF. (a) Liquid phase detection intensity of HMCA at different concentrations. (b) The linear relationship between the concentration of HMFCA and its liquid-phase detection intensity. (c) The liquid phase detection intensity curve corresponding to the HMFCA concentration at the intersection of the reverse extension line of the straight line in Figure b and the x-axis. (d) Liquid phase detection intensity of FFCA at different concentrations. (e) The linear relationship between the concentration of FFCA and its liquid-phase detection intensity. (f) The liquid phase detection intensity curve corresponding to the FFCA concentration at the intersection of the reverse extension line of the straight line in Figure e and the x-axis. (g) Liquid phase detection intensity of DFF at different concentrations. (h) The linear relationship between the concentration of DFF and its liquid-phase detection intensity. (i) The liquid phase detection intensity curve corresponding to the DFF concentration at the intersection of the reverse extension line of the straight line in Figure h and the x-axis.

Figure S10a and S10d represent the liquid-phase detection intensity curves at different concentrations of HMF and FDCA, respectively. Figure S10b and S10e show the linear relationship between the concentrations of HMF and FDCA and the liquid-phase detection intensity, respectively. Figure S10c and S10f show the liquid-phase detection intensity curves at the concentration where the linear relationship between the concentration and the detection intensity intersects the x-axis. In addition, Figure S11a, S11d, and S11g represent the liquid-phase detection intensity curves at different concentrations of HMFCA, FFCA, and DFF, respectively. Figure S11b, S11e, and S11h show the linear relationship between the concentrations of HMFCA, FFCA, and DFF and the liquid-phase detection intensity, respectively. Figure S11c, S11f, and S11i show the liquid-phase detection intensity curves at the concentration where the linear relationship intersects the x-axis. In Figures S10c, S10f, and S11c, S11f, S11i, no peaks are observed because the detected concentration corresponds to the intersection point of the extended line of the concentration-intensity linear relationship with the x-axis. At this point, the detection intensity of the corresponding peak is zero. Therefore, no peaks are observed in Figures S10c, S10f, and S11c, S11f, S11i, and this concentration represents the detection limit for the corresponding species.


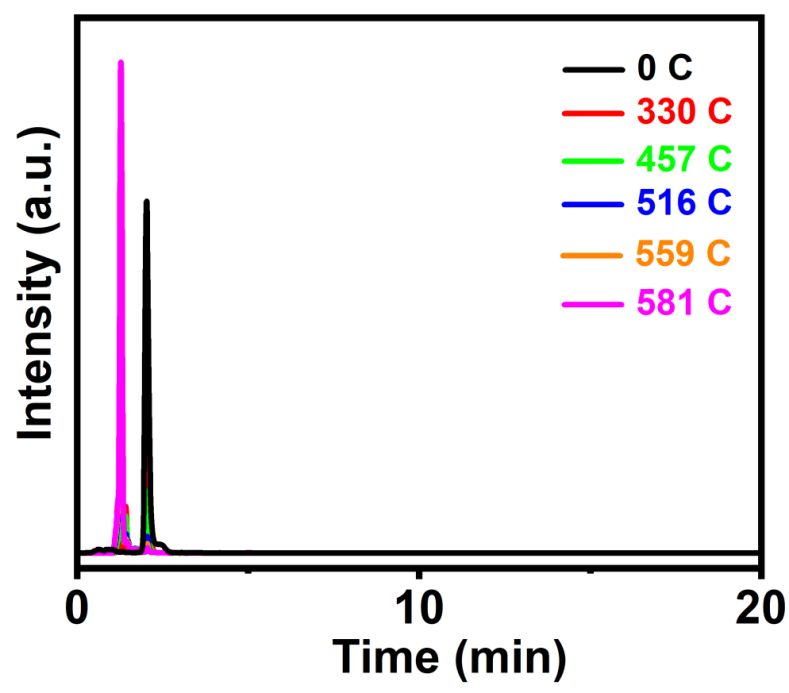


**Figure S12** The chromatogram of HMF oxidation reaction.

***
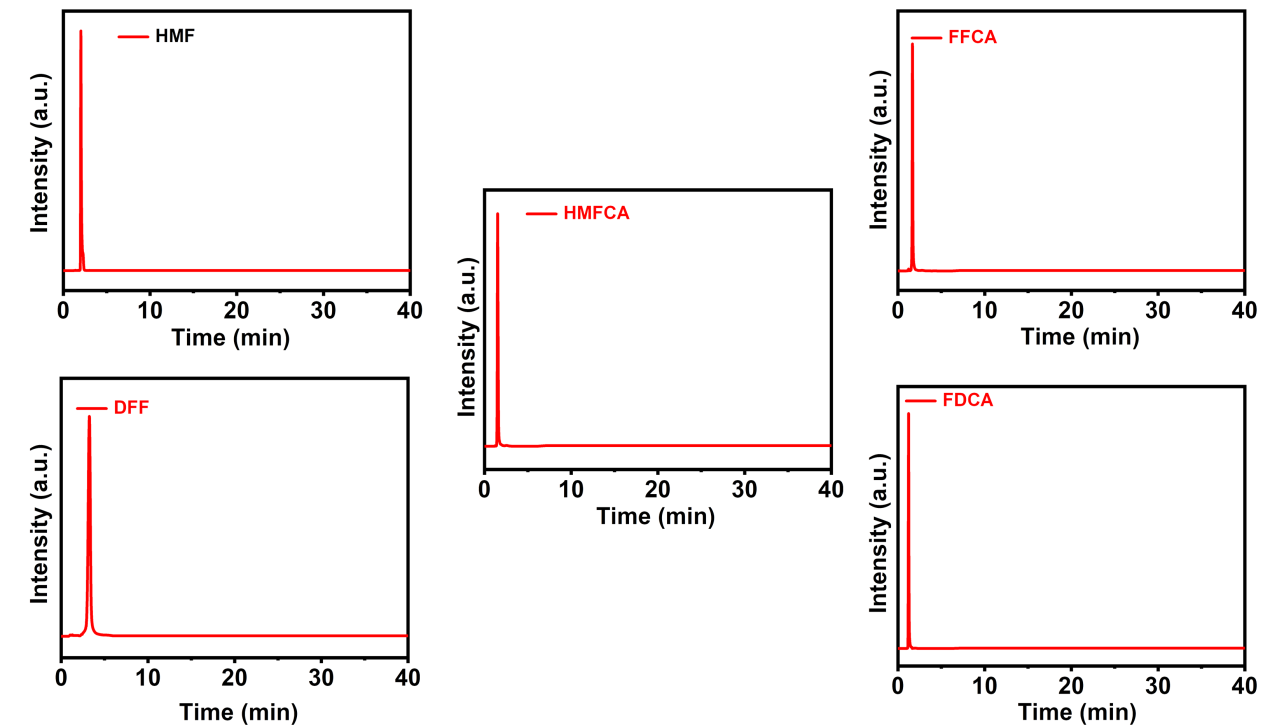
***

**Figure S13** The HPLC measurement curves of pure HMF, HMFCA, FFCA, FDCA, and DFF.


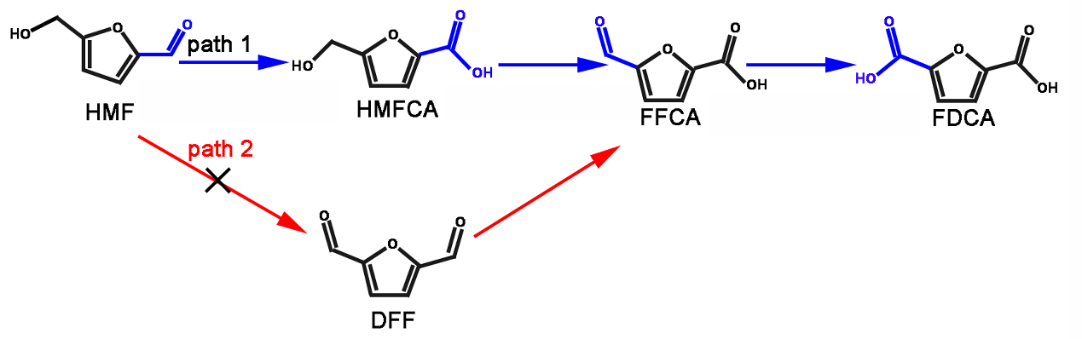


**Figure S14** The oxidation path of HMF for the V-CoP catalyst.


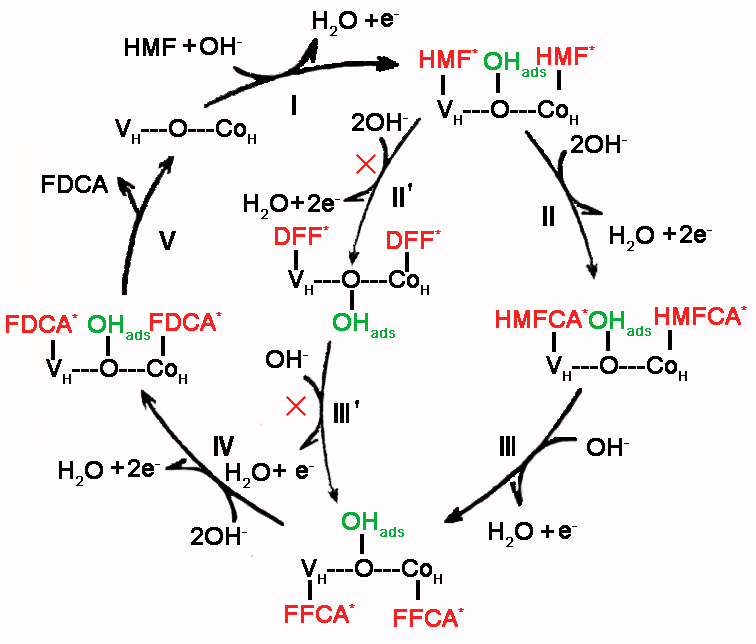


**Figure S15** Possible HMF oxidation pathways of V-CoP catalyst.


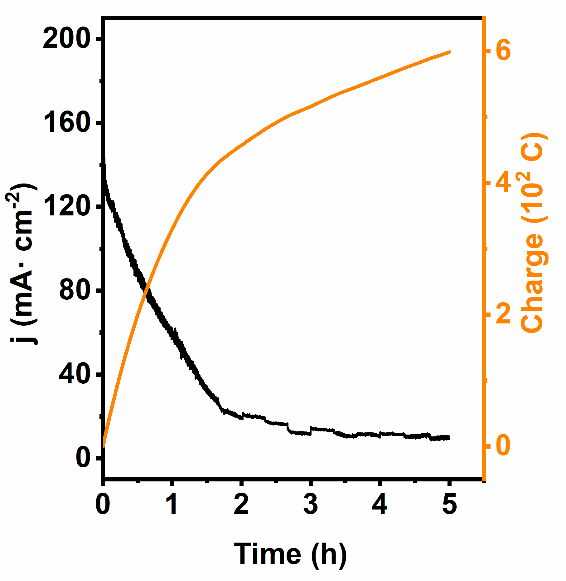


**Figure S16** The I-t curve of V-CoP at constant potential of 1.38 V in 1.0 M KOH with 100 mM HMF via passing the charge of 581 C.

*
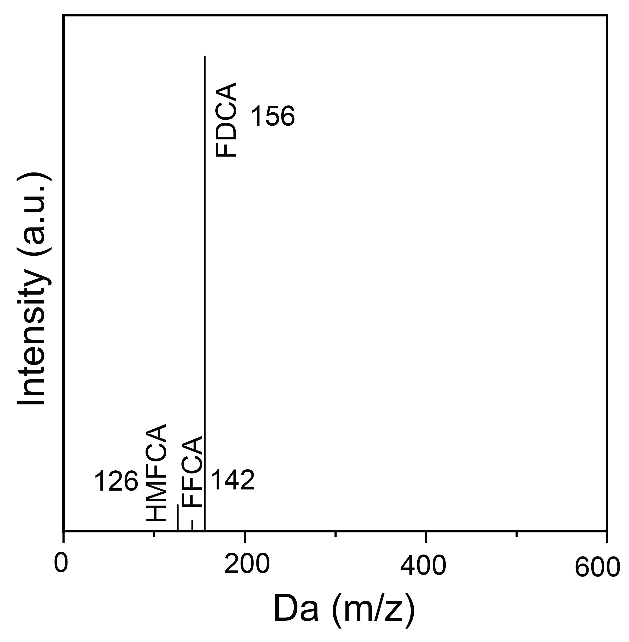
*

**Figure S17** Mass spectrometry (MS) results of FDCA and its by-products in negative ion mode.

**Table S2** Comparison HMFOR property of V-CoP with other electrocatalysts in 1.0 M KOH with 100 mM HMF.

| **Samples** | **Oxidation Potential (V vs RHE)** | **HMF Conversion (%)** | **FDCA Selectivity (%)** | **Faraday efficiency (%)** | **HMF concentration (mM)** | **Reference** |
| --- | --- | --- | --- | --- | --- | --- |
| Pt/Ni(OH)_2_ | 1.50 | ~100 | ~100 | 98.7 | 50 | *Angew. Chem. Int. Ed.* **2021***, 60*, 22908-22914 |
| MoO_2_- FeP@C | 1.45 | 99.4 | 98.6 | 97.8 | 10 | *Adv. Mater.***2020***,* 32, 2000455 |
| CoP-CoOOH | 1.42 | 98.3 | 96.3 | 96.3 | 150 | *Appl. Catal. B-Environ‌‌* **2022***,* 315, 121588 |
| NiO-Co_3_O_4_ | 1.45 | ~100 | 98 | 96 | 10 | *Sci. China. Chem.* **2020***,* 63, 980-986 |
| CuCo_2_O | 1.45 | ~100 | 93.7 | 94 | 50 | *Angew. Chem. Int. Ed.* **2020**, *59*, 19215-19221 |
| (PdAu)_7_ | 0.82 | ~ | 17.5 | 85.8 | 10 | *ACS Nano* **2020**, 14, 6812-6822 |
| NiCoP | 1.46 | 98.7 | 98.8 | 95.8 | 300 | *J. Mater. Chem. A* **2021**, *9*, 18421-18430 |
| CoMoP | 1.36 | 99.9 | 95.8 | 93.0 | 100 | *Appl. Catal. B-Environ* **2024***, ‌‌340*, 123249 |
| V_o_-NiO | 1.42 | 99.7 | 99.2 | 85.7 | 100 | *Chem. Eng. J.* **2022***,* **444**, 136693 |
| CoOOH | 1.42 | ~100 | ~100 | ~100 | 100 | *Green Chem.* **2021**, *23*, 2525-2530 |
| CoOOH/Ni | 1.50 | 86.3 | 85.5 | 90.2 | 100 | *Appl. Catal. B-Environ* **2021,** *297*, 120396 |
| Cu_x_S@NiCoLDH | 1.32 | ~100 | 99.0 | 99.0 | 10 | *J. Mater. Chem. A* **2020***, 8*, 1138-1146 |
| V-CoP | 1.38 | 99.6 | 97.8 | 96.7 | 100 | This work |

**Table S3** ICP-MS measurement of V content in electrolyte before and after HMF catalytic oxidation over V-CoP/NF.

|  | Initial (μg/kg) | After 100.0 h (μg/kg) |
| --- | --- | --- |
| V | 39519.2926045016 | 39624.115755627 |

*
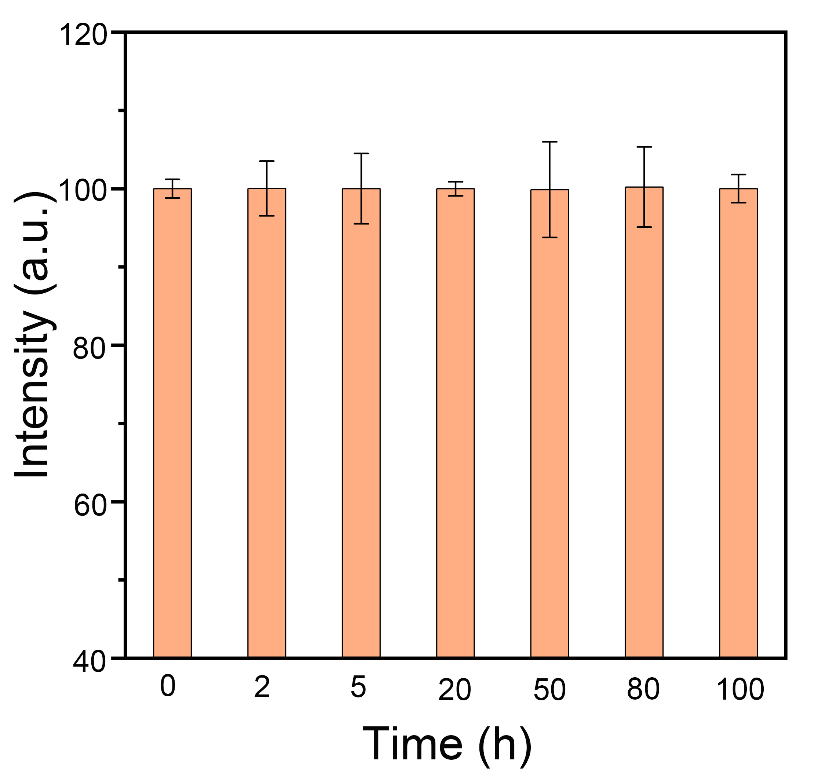
*

**Figure S18** An I-T test was performed on the activated V-CoP/NF at 1.38 V, and the V-*O signal intensity was recorded at different time points. The error bar represents the standard deviation of four independent measurements (n=4, mean ± SD).

**Table S4** The comparison of the performance and stability of V-CoP/NF and previously reported HMF electrocatalysts in alkaline media.

| Catalyst | Potential | cycles | HMF concentration (mM) | Electrolyte | Ref |
| --- | --- | --- | --- | --- | --- |
| ZIF-CoNi | 1.35 | 7 | 10 | 1.0 M KOH | *Adv. Energy Mater.* **2024**, 2405364 |
| Pt/CuO@CF | 1.3 | 15 | 10 | 1.0 M KOH | Adv. Mater. **2025**, 2417684 |
| Pd/NiCo | 1.32 | 5 | 10 | 1.0 M KOH | *Angew. Chem. Int.*  *Ed.* **2025***,* 135, e202311696 |
| NiCo(OH)_x_ | 1.2 | 5 | 50 | 1.0 M KOH | *Angew. Chem. Int.*  *Ed.* **2024***, 136*, e202408109 |
| 1J85-laser | 1.4 | 15 | 50 | 1.0 M KOH | *Adv. Sci.* **2023,** *10*, 2302641 |
| Ru1-NiO | 1.283 | 5 | 50 | 1.0 M PBS | *Angew. Chem. Int.*  *Ed.* ***2022,*** 134, e202200211 |
| F-NiCo_2_O_4_ | 1.35 | 4 | 50 | 1.0 M KOH | *Chem. Eng. J.* **2023**, *457*, 141344 |
| NiFeMoB | 1.35 | 3 | 30 | 1.0 M KOH | *Angew. Chem. Int. Ed.* **2025***,* e202424345 |
| β-NiOOH | 1.26 | 5 | 50 | 1.0 M KOH | *Green Chem.***2025***, 27*, 2117 |
| Ni8PBA | 1.36 | 6 | 50 | 1.0 M KOH | J. Am. Chem. Soc. **2025**, *147*, 8832−8840 |
| A-Co-Ni_2_P | 1.3 | 8 | 100 | 1.0 M KOH | *Nat. Commun.* **2024**, 15, 8072 |
| NiCo PBA | 1.36 | 10 | 20 | 1.0 M KOH | *ACS Catal.* **2024**, *14*, 9565−9574 |
| FeP-NiMoP_2_/FNF | 1.333 | 10 | 10 | 1.0 M KOH | *Nat. Commun.* **2022**, *13*, 3125 |
| V-CoP/NF | 1.131 | 20 | 100 | 1.0 M KOH | This work |


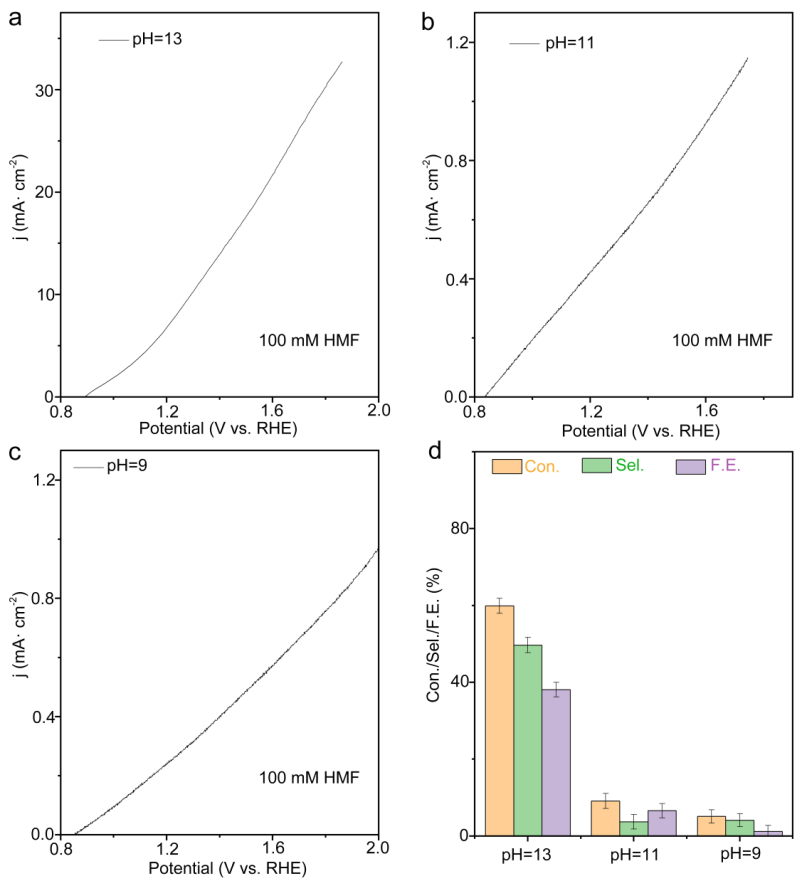


**Figure S19** (a-c) The polarization curves. (d) HMF conversion rates, FDCA selectivity, and Faraday efficiency of V-CoP at different pH values. The error bar represents the standard deviation of four independent measurements (n=4, mean ± SD).


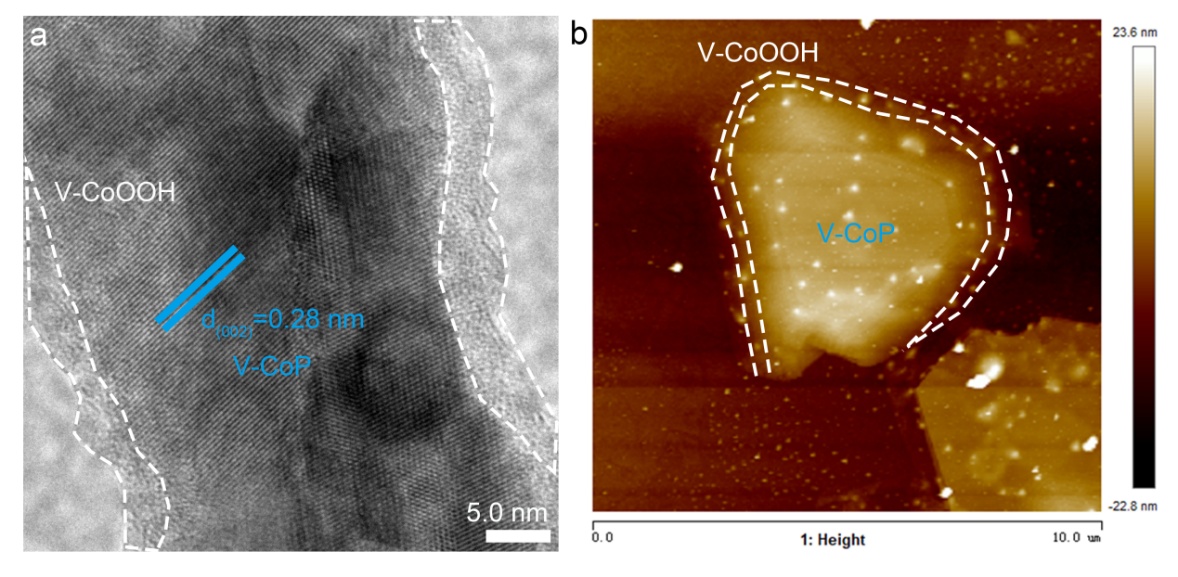


**Figure S20** (a) The HRTEM of V-CoP/V-CoOOH formation from V-CoP after HMFOR. (b) The atomic force microscope (AMF) high-profile of V-CoP/V-CoOOH.


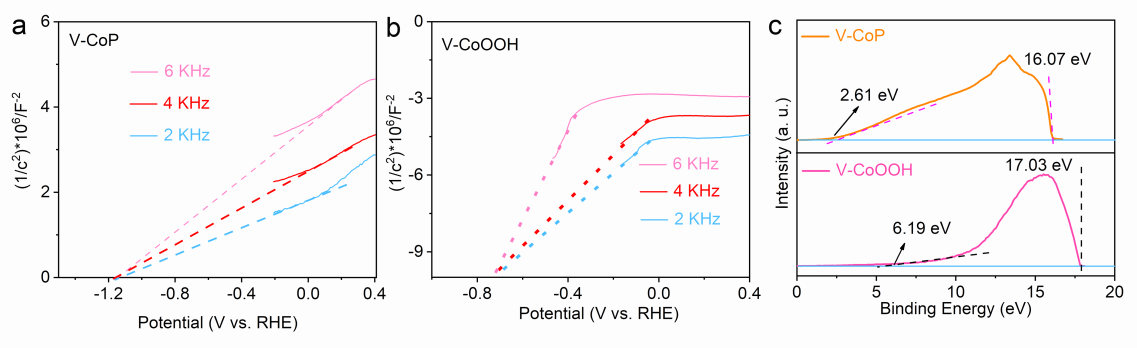


**Figure S21** (a) Mott–Schottky (MS) plots of V-CoP and (b) V-CoOOH. (c) Ultraviolet photoelectron spectroscopy of V-CoP (orange line) and V-CoOOH (pink line), which can determine the valance band position of V-CoP and V-CoOOH. The flat band potential (E_fb_) for V-CoP and V-CoOOH are obtained by Mott–Schottky (MS) plots.


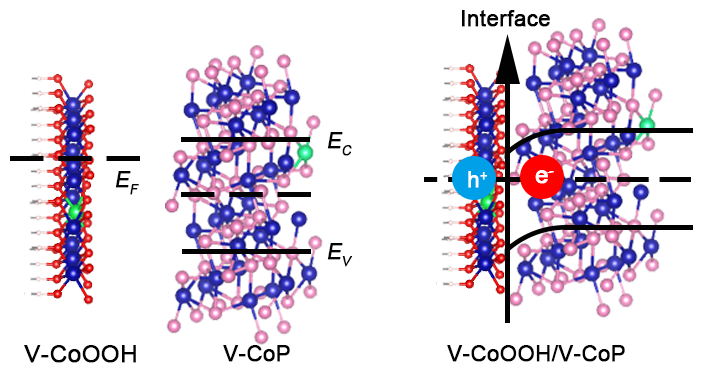


**Figure S22** Schematic structures of V-CoOOH and V-CoP and the epitaxial relationships after integration of the tow components together as Schottky heterojunctions, resulting in electron-rich (red) and electron-deficient (blue) areas at the contact interface.


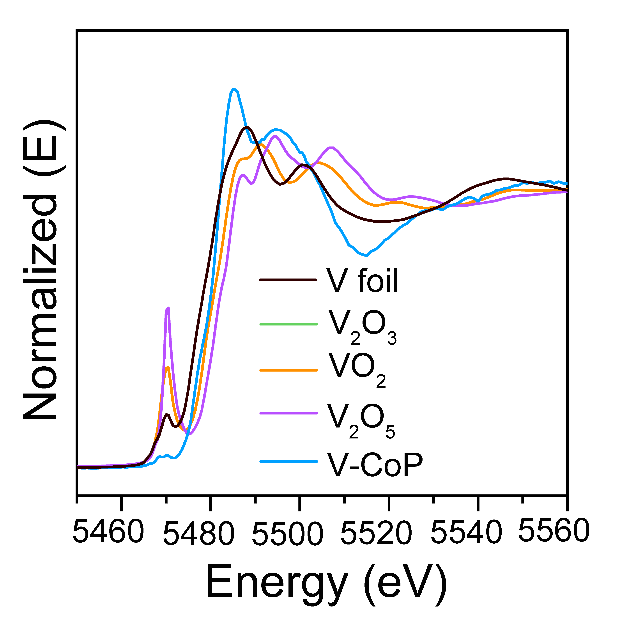


**Figure S23** XANES spectra at the V K-edge.


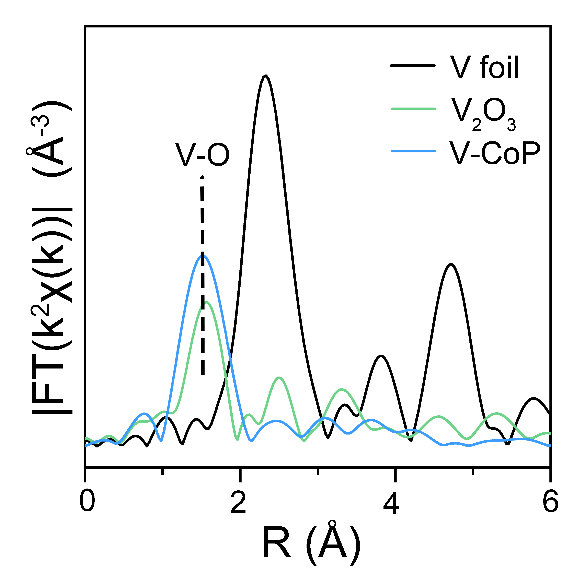


**Figure S24** FT of V K-edge extended X-ray absorption fine structure (EXAFS) of V-CoP.


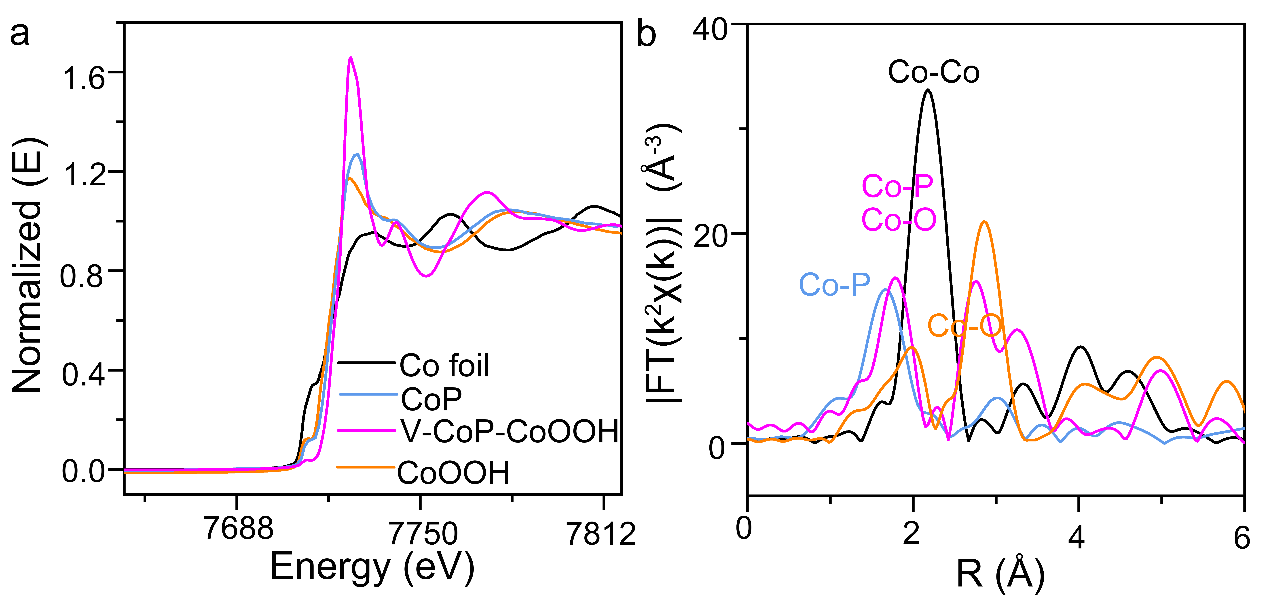


**Figure S25** XANES spectra at the (a) Co K-edge. (b) FT of the Co K-edge EXAFS of catalysts.


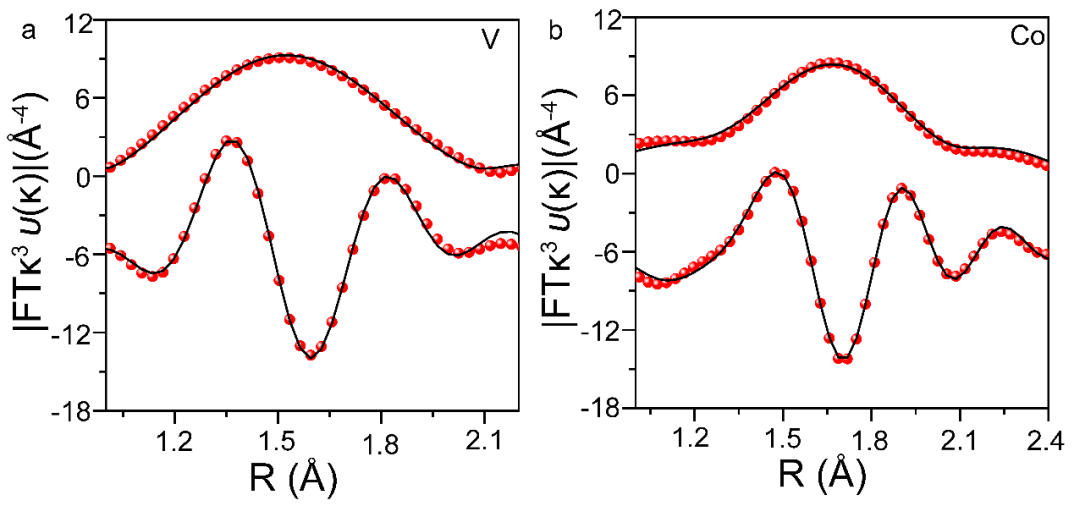


**Figure S26** After HMF: (a) V K-edge FT-EXAFS fitting curves.(b) Co K-edge FT-EXAFS fitting curves.

**Table S5** Structural parameters from Co K-edge EXAFS fitting for reconstructed V-CoP.

| sample | Path | R (Å) | N | σ^2^ (Å^2^) | ΔE_0_ (eV) | R-factor (%) | ΔR(Å) |
| --- | --- | --- | --- | --- | --- | --- | --- |
| Reconstructed V-CoP | Co-P | 1.65 | 2.30 | 0.0035 | -1.02 | 0.51 | -0.09 |
|  | Co-O | 2.2 | 1.87 | 0.008 | -1.02 | 0.51 | 0.02 |

Fitting range: 1.0 ≤ R (Å) ≤ 2.4.

**Table S6** Structural parameters from V K-edge EXAFS fitting for reconstructed V-CoP.

| sample | Path | R (Å) | N | σ^2^ (Å^2^) | ΔE_0_ (eV) | R-factor (%) | ΔR(Å) |
| --- | --- | --- | --- | --- | --- | --- | --- |
| Reconstructed V-CoP | V-O | 2.47 | 2.39 | 0.006 | -1.02 | 0.51 | -0.07 |

Fitting range: 1.0 ≤ R (Å) ≤ 2.2.

**Note:** R, distance between absorber and backscatter atoms, N, coordination number, σ^2^, Debye-Waller factor to account for both thermal and structural disorders, ΔE_0_, inner potential correction, R-factor (R_f_, %) indicates the goodness of the fit. Bold value shows coordination number derived from the crystal structures. S_0_^2^ is fixed to 0.8. Error bounds (accuracies) that characterize the structural parameters obtained by EXAFS spectroscopy are estimated as R ± 1%, N ± 30%, σ^2^ ± 20%, ΔE_0_ ± 20%.


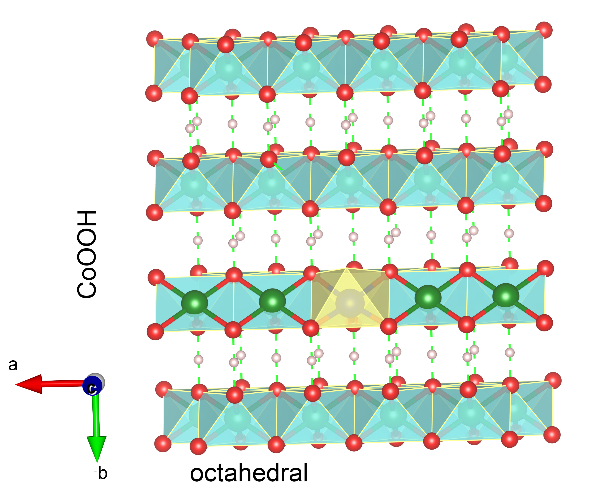


**Figure S27** The crystal structure of CoOOH.

**
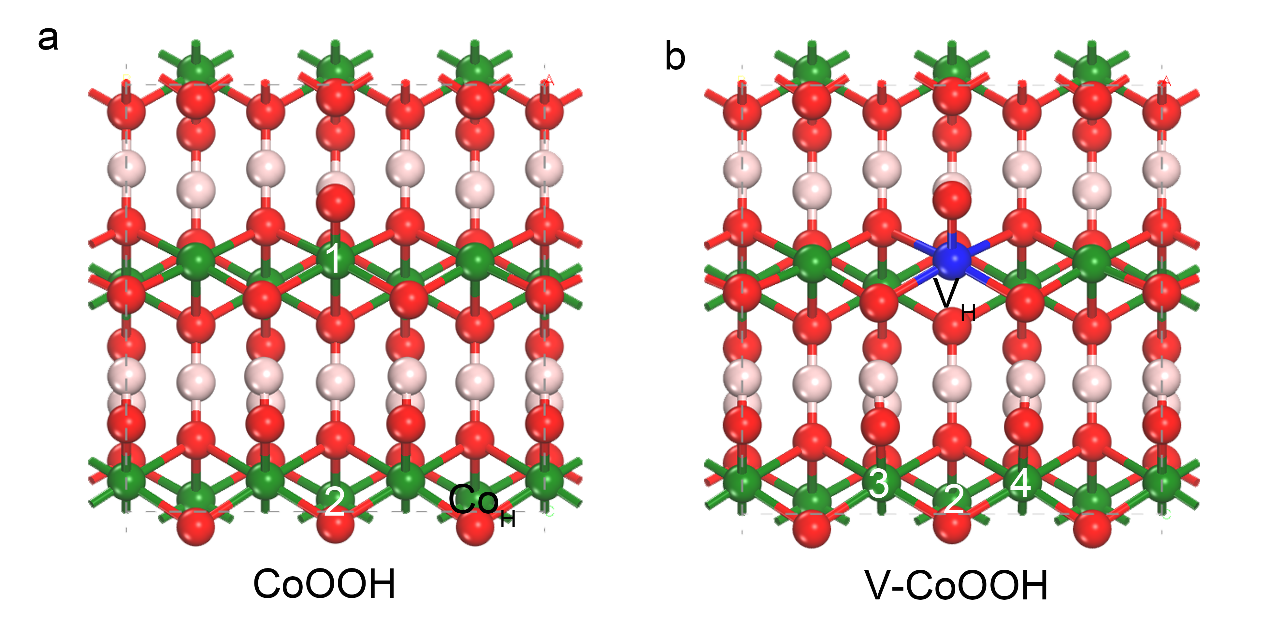
**

**Figure S28** Theoretical structural models of CoOOH and V-CoOOH.

**Table S7** For the substitution at position 2, the values for positions 3 and 4 were calculated using differential charge density.

| Position/Element | 2 | 3 | 4 |
| --- | --- | --- | --- |
| Co _CoOOH_ | 1.13 | 1.14 | 1.14 |
| V _V-CoOOH_ | 1.35 | / | / |
| Co _V-CoOOH_ | / | 1.15 | 1.15 |

*
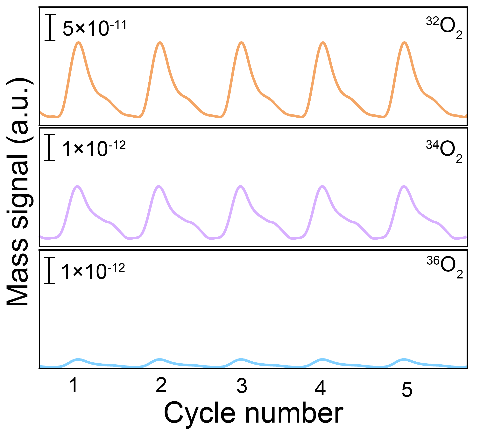
*

**Figure S29** DEMS signals of O_2_ products for V-CoP/NF in the electrolyte using H_2_^18^O as the solvent during three times of LSV in the potential range of 1.17–1.72 V versus RHE, with a 10 mV/s scan rate.


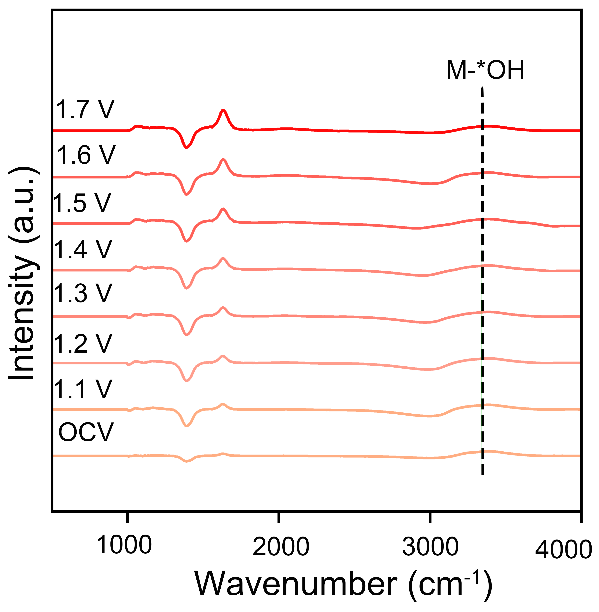


**Figure S30** Electrochemical in situ IR spectra (500-4000 cm^-1^) of HMFOR with V–CoP/NF in 0.1 M HMF+1.0 M KOH.

*
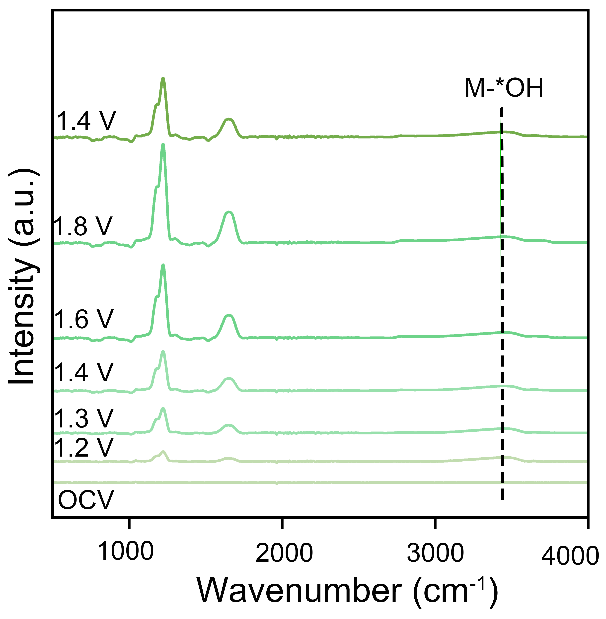
*

**Figure S31** Electrochemical in situ IR spectra (500-4000 cm^-1^) of HMFOR with V–Ni_2_P/NF in 0.1 M HMF+1.0 M KOH.


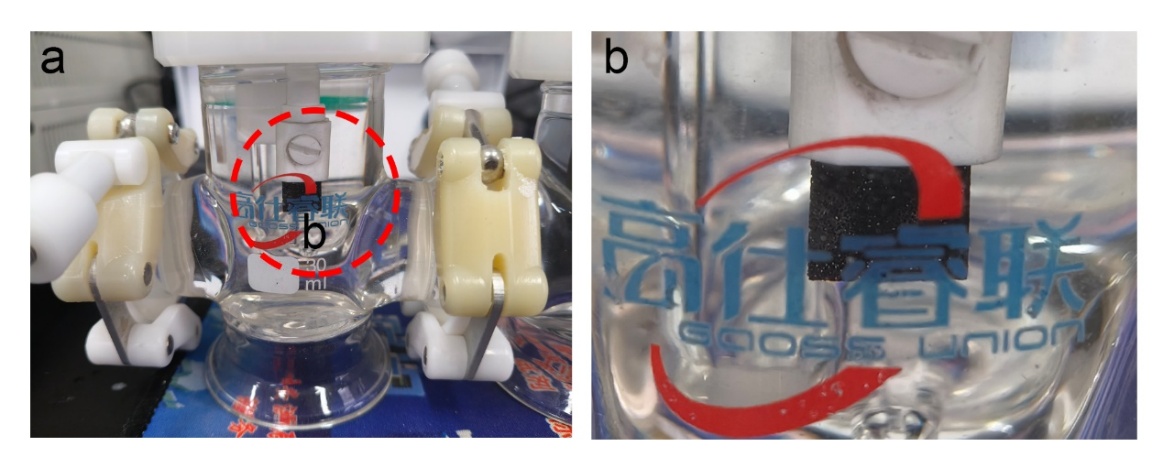


**Figure S32** After adding HMF, no significant gas formation was observed at a voltage of 1.6 V vs. RHE.

**
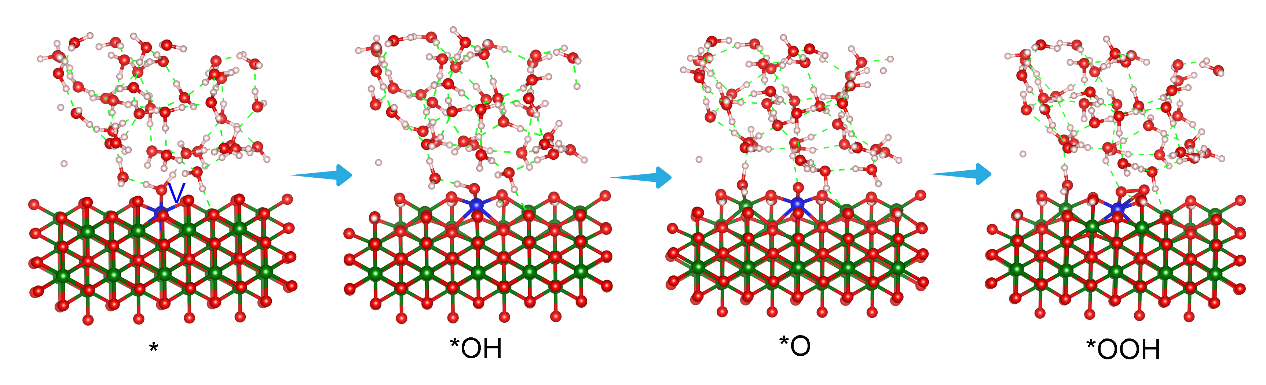
**

**Figure S33** Catalytic structures of each stage corresponding to the indirect catalytic mechanism for HMF mechanism under solvent effects.

**
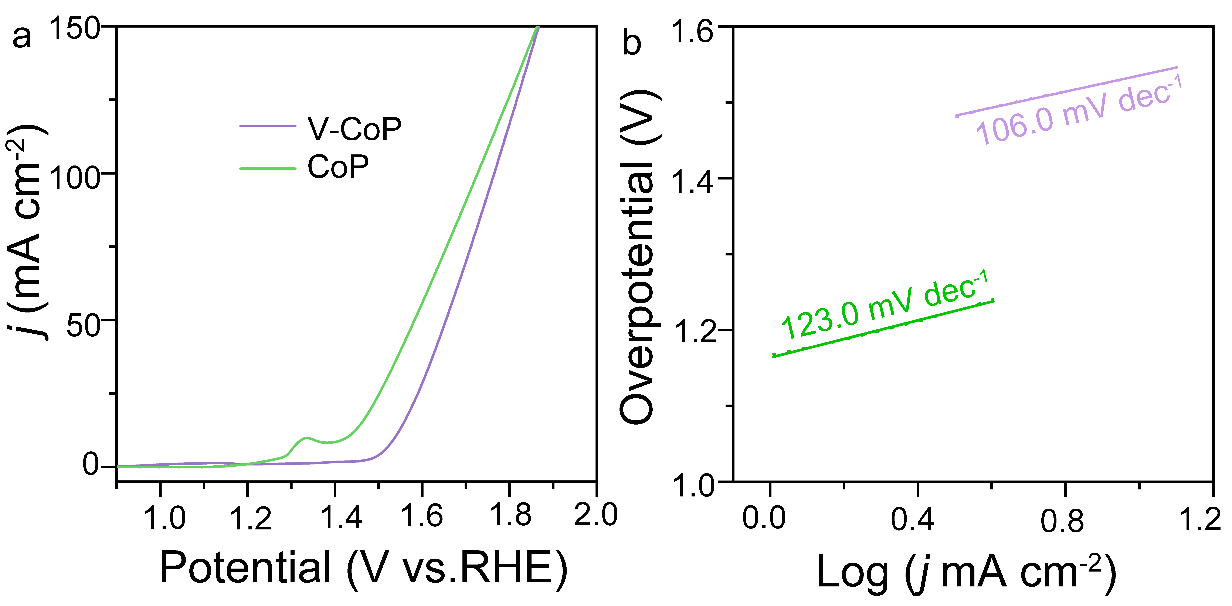
**

**Figure S34** Electrochemical HMF performances in 1 M KOH solution. (a) Polarization curves of V-CoP/NF and CoP/NF. (b) Tafel slopes of V-CoP/NF and CoP/NF.

*
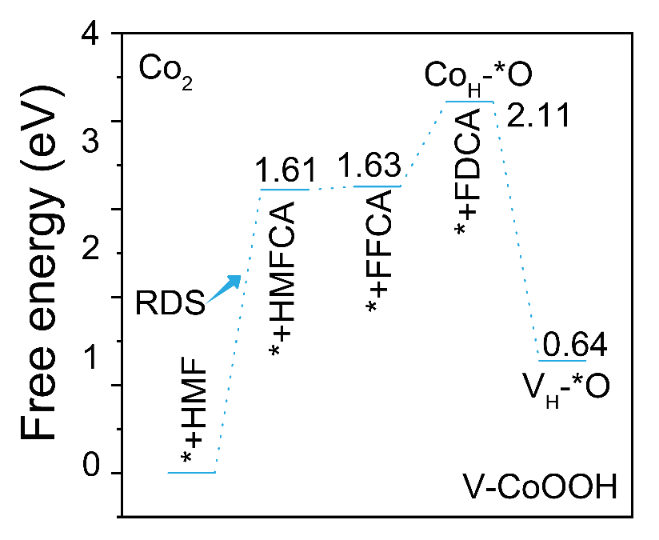
*

**Figure S35** Free energy diagrams for the catalytic conversion of HMF at the V_H_ sites (V doped Co2 sites) corresponding to M-*O in the Co_H_-O-V_H_ model of V-CoOOH.

**
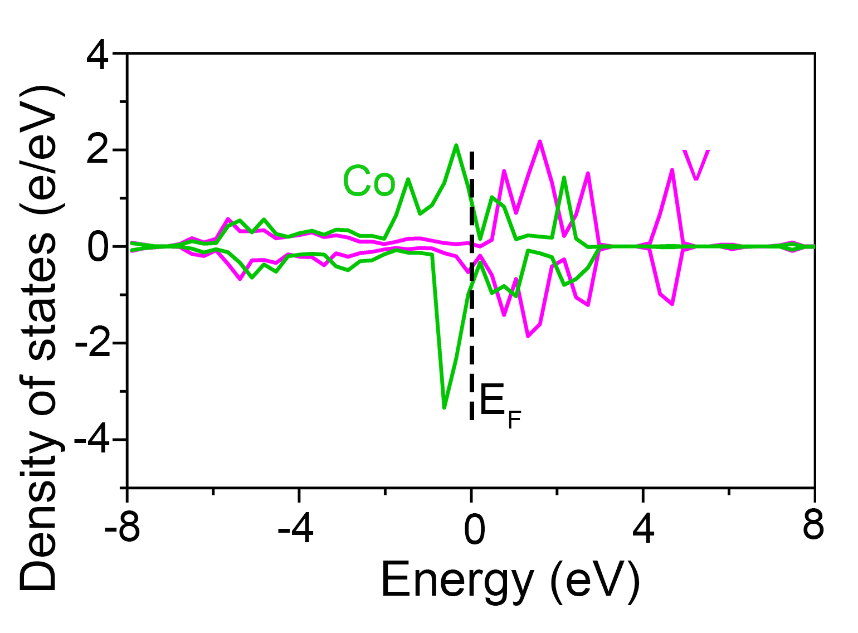
**

**Figure S36** Projected density of states of Co 3d and V 3d. Fermi level is set to zero.

**
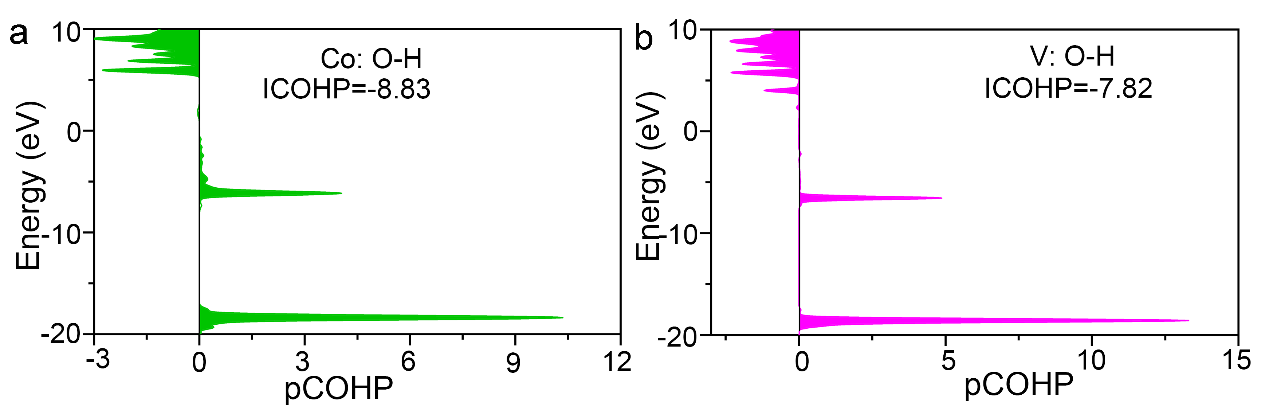
**

**Figure S37** COHP of (a) Co-O and (b) V-O bonds in V-CoOOH.

**
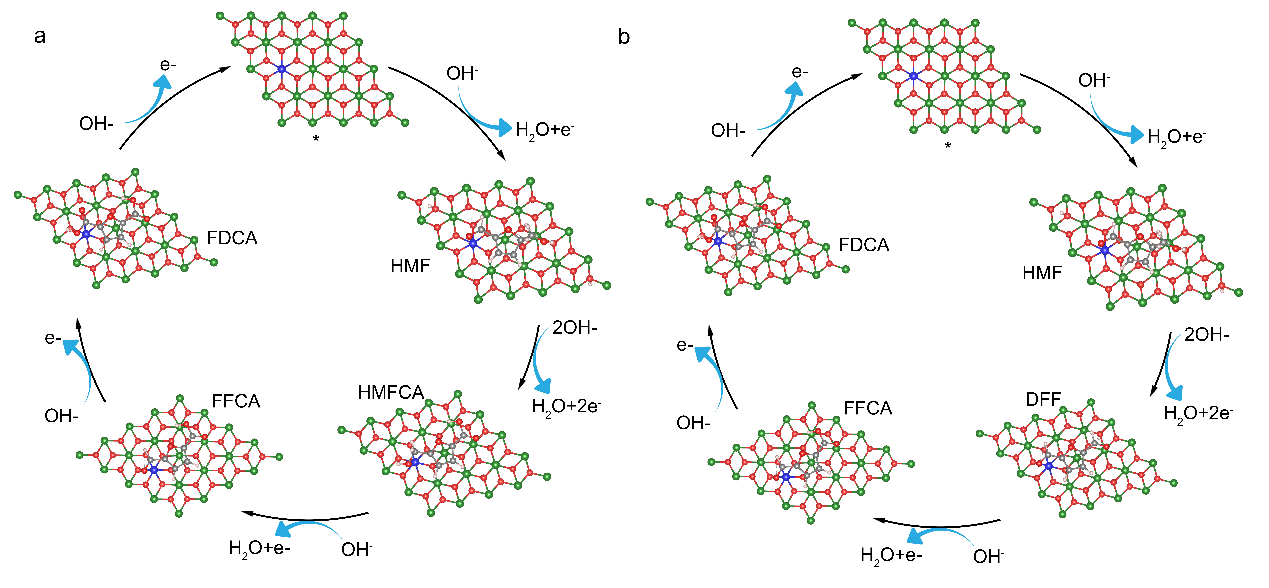
**

**Figure S38** The schematic diagram of different active sites (a) V and (b) Co directly adsorbing and catalyzing the HMF mechanism.

**
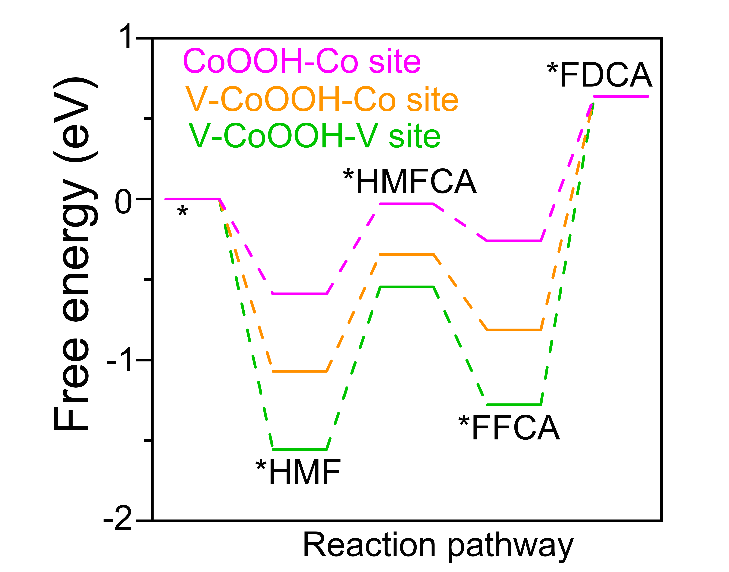
**

**Figure S39** The free energy diagram of direct adsorption and continuous catalysis of HMF at V and Co sites in the V-CoOOH and CoOOH models.

**
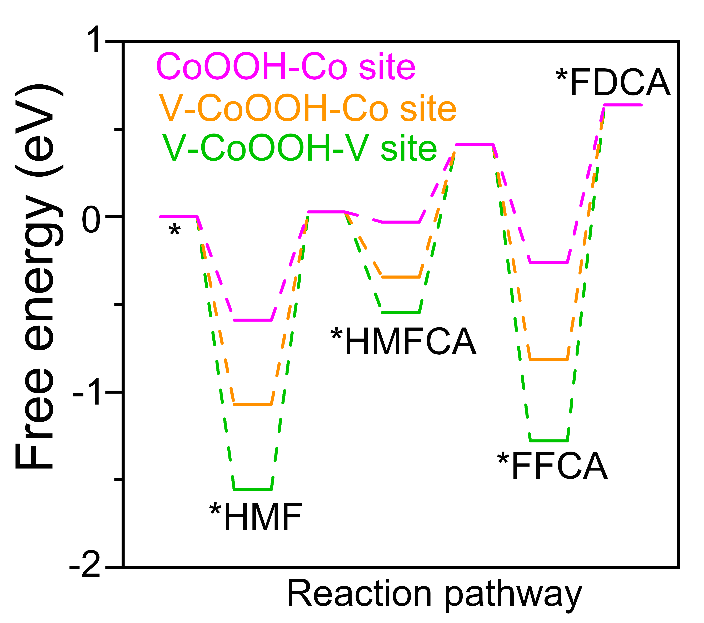
**

**Figure S40** The free energy diagram of direct adsorption and non-continuous catalysis of HMF at V and Co sites in the V-CoOOH and CoOOH models.


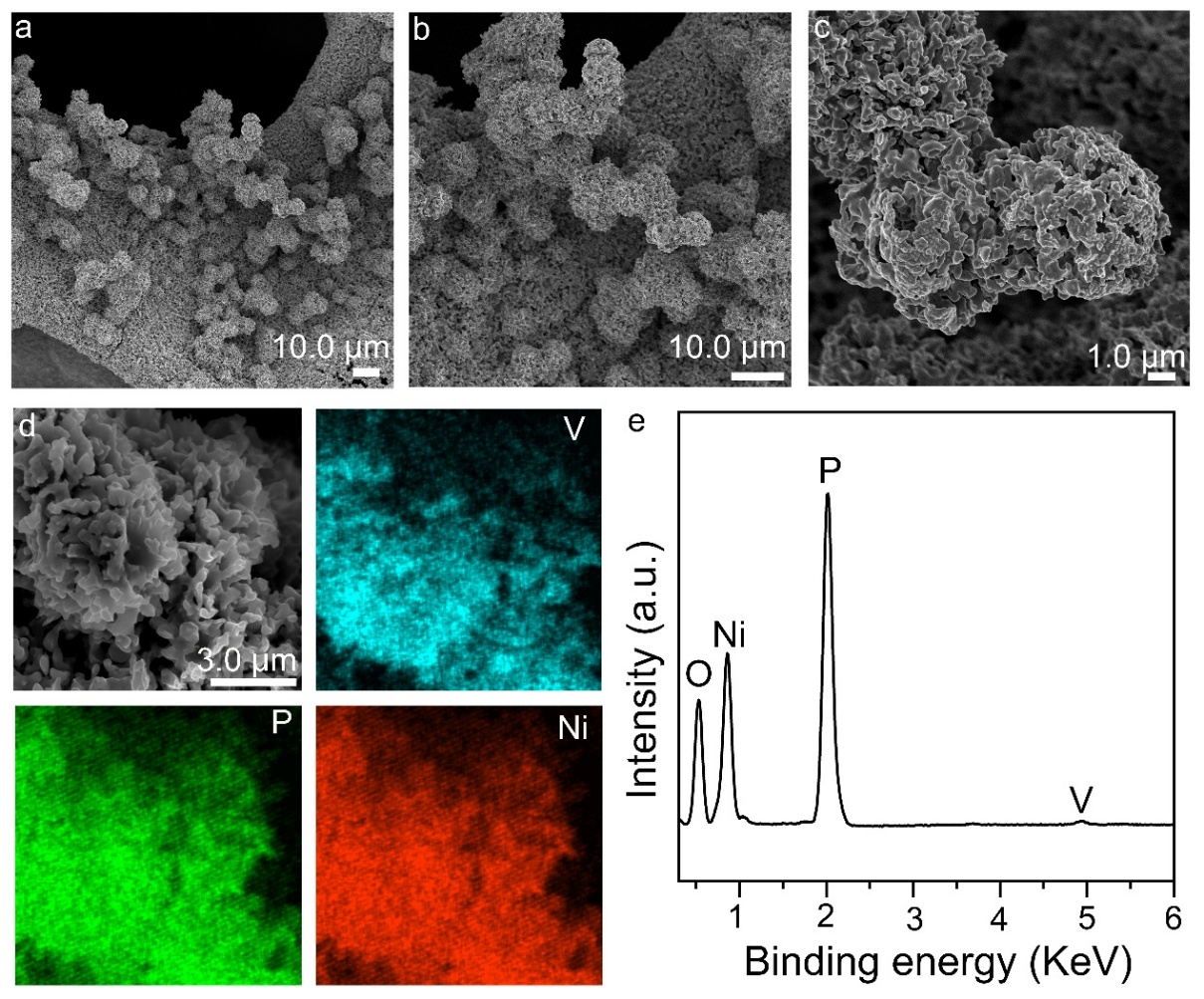


**Figure S41** V-Ni_2_P/NF: SEM images at different scales. (a, b) 10 μm. (c) 1.0 μm. (d) EDS-mapping. (e) EDS spectrum.


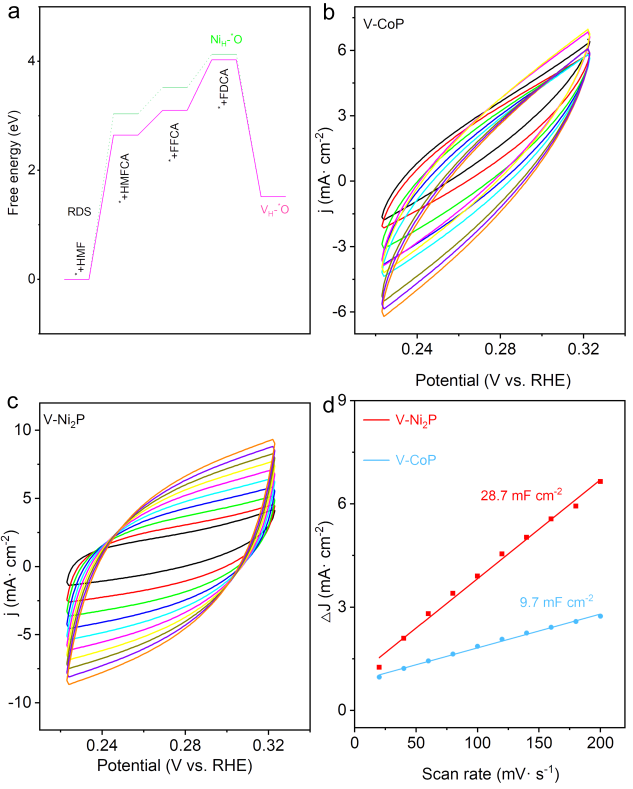


**Figure S42** (a) Free energy diagrams for the catalytic conversion of HMF at the V_H_ and Co_H_ sites corresponding to M-*O in the Ni_H_-O-V_H_ model of V-Ni_2_P. (b) Cyclic voltammograms of V-CoP and (c) V-Ni_2_P. (d) Double-layer capacitance diagrams of V-CoP and V-Ni_2_P.


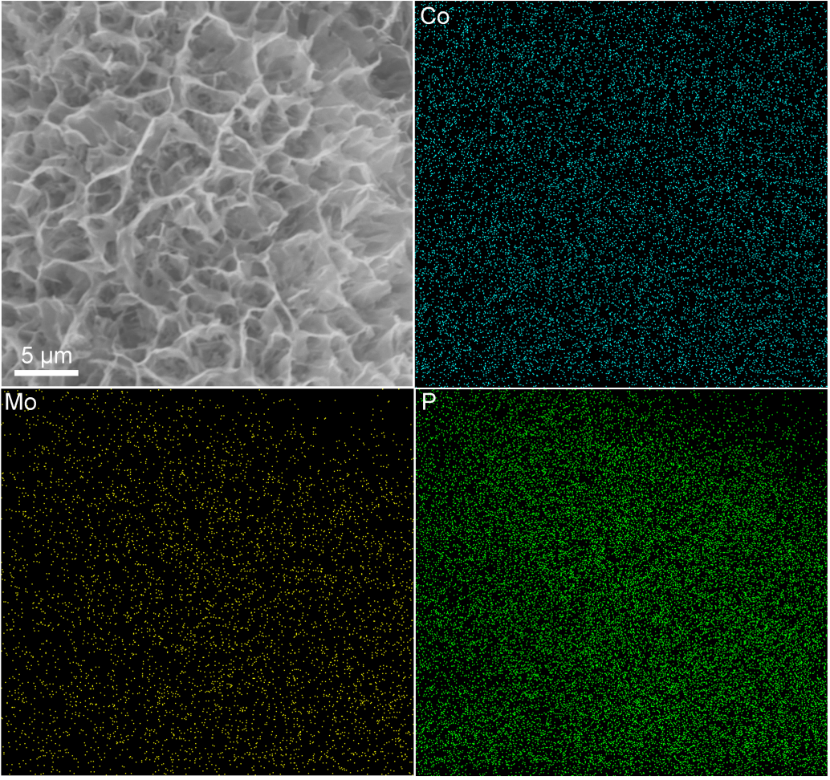


**Figure S43** The SEM images elements mapping of Mo-doped CoP.


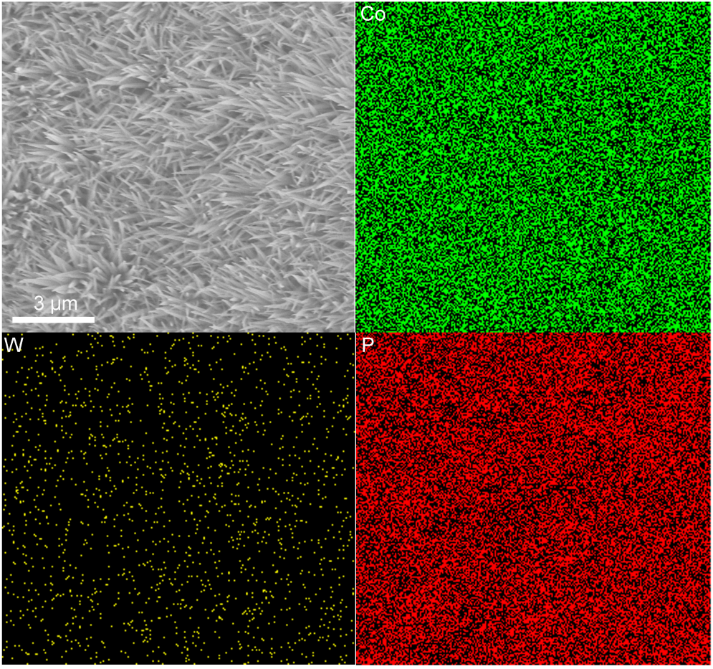


**Figure S44** The SEM images elements mapping of W-doped CoP.


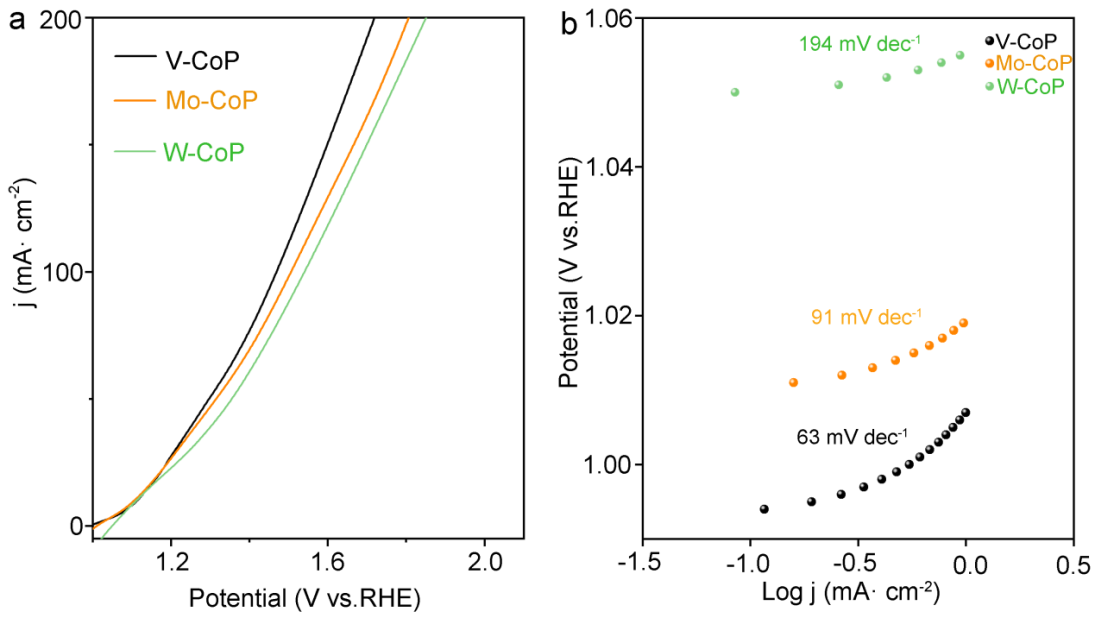


**Figure S45** (a) The polarization curve and (b) Tafel slope of V/Mo/W-doped CoP.


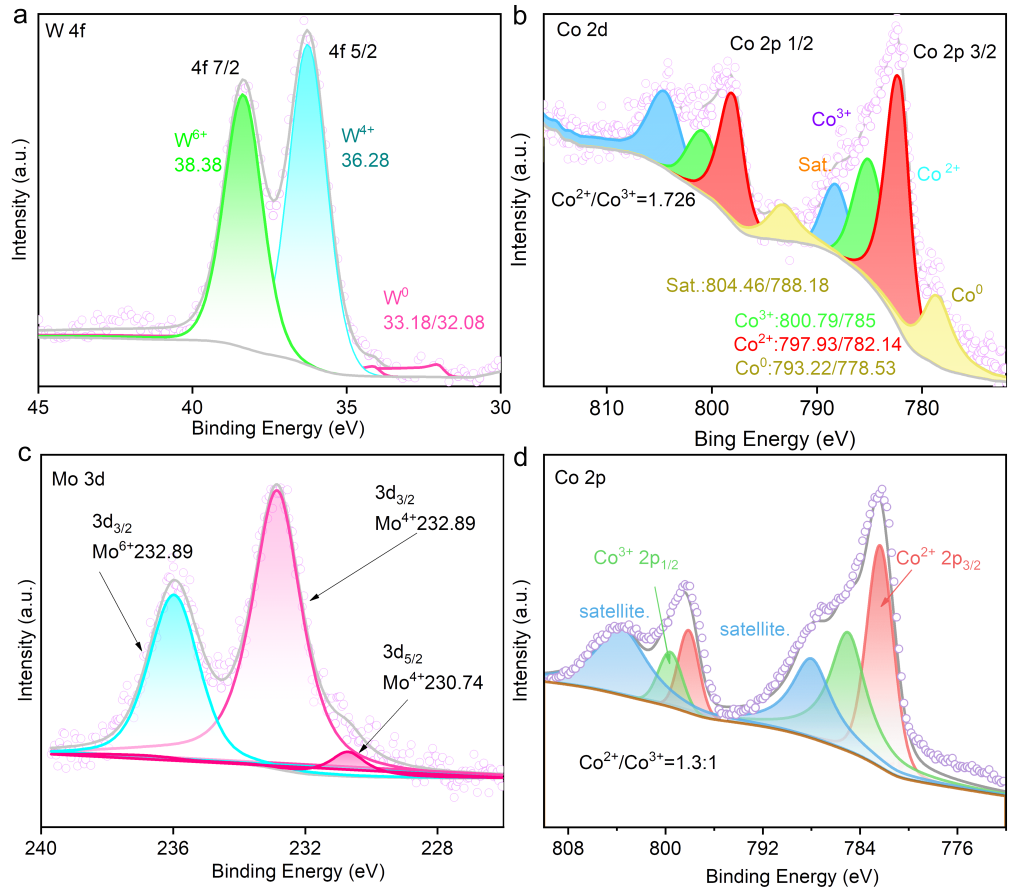


**Figure S46** (a) The W 4f and (b) Co 2p spectra of W-doped CoP XPS. (c) The Mo 3d and (b) Co 2p spectra of Mo-doped CoP XPS.


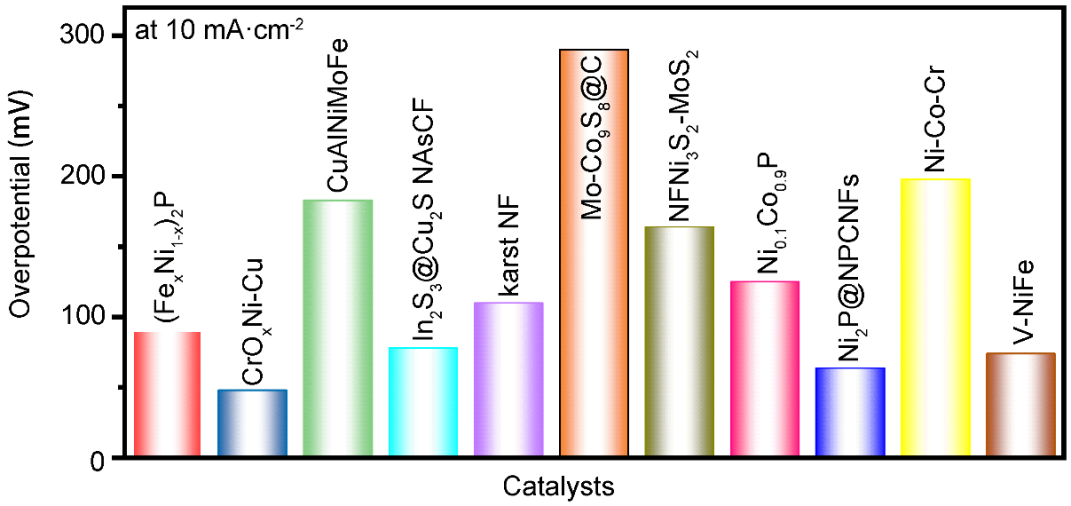


**Figure S47** HER activity comparison of V-CoP/NF and reported catalysts ((Fe_x_Ni_1-x_)_2_P (*Nano Energy* **2019**, *56*, 813-822), CrO_x_Ni-Cu (*Nat. Energy* **2019**, *4*, 107-114), CuAlNiMoFe (*Adv. Functional Mater.* **2021**, *31*, 2009613), In_2_S_3_@Cu_2_SNAsCF (*Journal of Materials Chemistry A* **2023**, *11*, 2262-2272), karst NF (*Energ. Environ. Sci.* **2020**, *13*, 174-182), Mo-Co_9_S_8_@C (*Adv. Energy Mater.* **2020**, *10*, 1903137), NFNi_3_S_2_-MoS_2_ (*Small* **2022**, *18*, 2201896), Ni_0.1_Co_0.9_P (*Angew. Chem. Int. Ed.* **2018**, *57*, 15445-15449), Ni_2_P@NPCNFs (*Angew. Chem. Int. Ed.* **2018**, *57*, 1963-1967), Ni-Co-Cr (*Nano Res.* **2019**, *12*, 1431-1435), V-NiFe (*Small*, **2024**, 2310642).


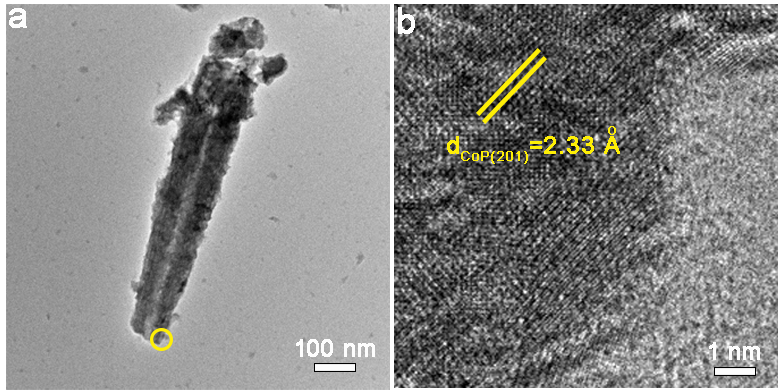


**Figure S48** (a) TEM and (b) HRTEM of V-CoP after HER testing.


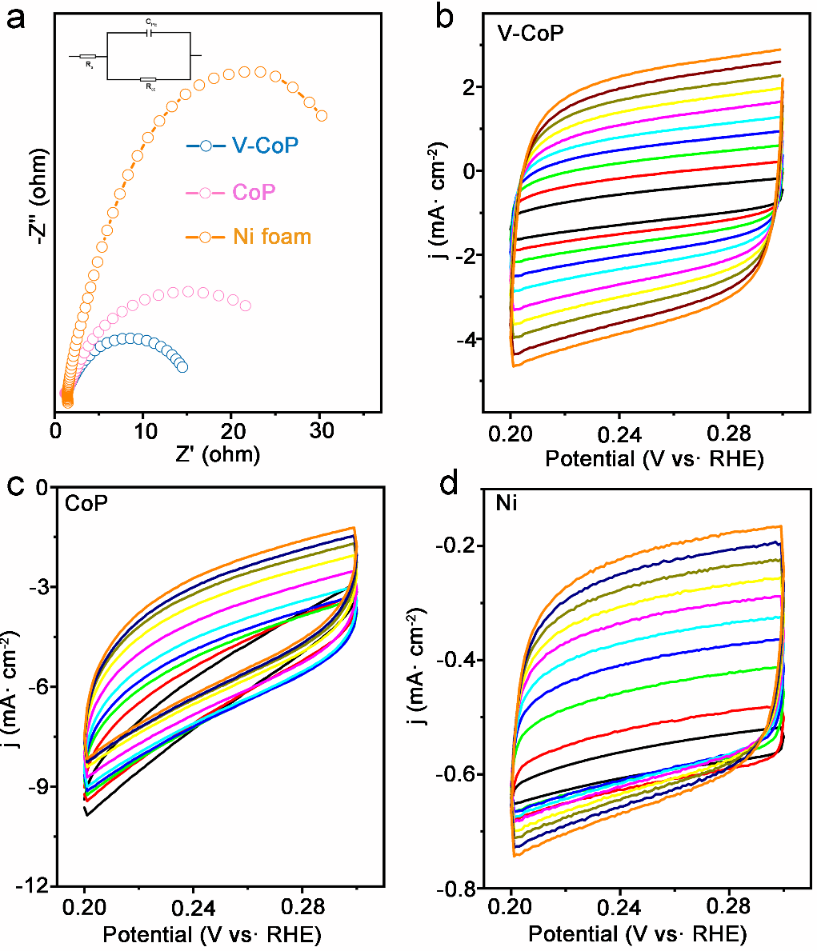


**Figure S49** (a) Nyquist plots of catalysts (Inset: The equivalent circuit of electrochemical impedance spectroscopy for V-CoP/NF). (b-d) The cyclic voltammetry curves of catalysts with scanning rates from 20 to 200 mV s^-1^.


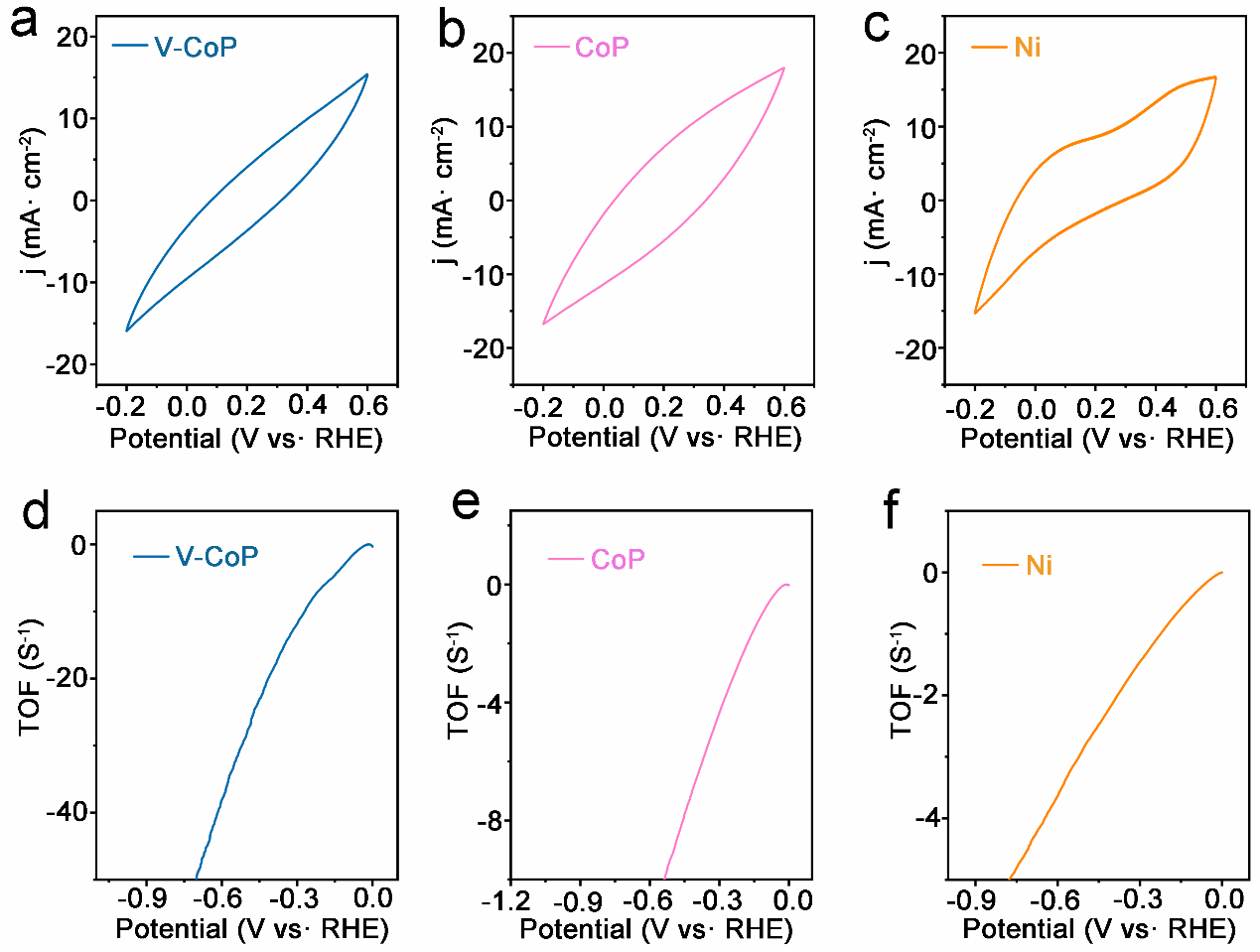


**Figure S50** The cyclic voltammetry curves of V-CoP, CoP, Ni foam are measured in the voltage range of -0.2 to 0.6 V vs. RHE at pH = 7.0 with a scan rate 50 mV s^-1^. The polarization curves and TOFs of catalysts are obtained at pH = 1.0.

Impedance spectra (EIS) (Figure S48a) and (Figure S48a, inset) circuit diagram shows the resistance (*R_ct_*) of V-CoP/NF is smaller than that of CoP/NF and NF, demonstrating the V-doped improved CoP charge conduction. Since electrochemical surface area (ECSA) is proportional to the double layer capacitance (*C_dl_*) value, it can be evaluated by testing the *C_dl_*. Figure S48b-d shows the *C_dl_* of V-CoP/NF (15.1 mF cm^−2^) is superior to CoP/NF (5.6 mF cm^−2^) and NF (1.1 mF cm^−2^), illustrating the V-CoP/NF possess larger surface area compare with other catalysts. The turnover frequency (TOF) value of V-CoP/NF (2.46 s^-1^) at 100 mV in neutral medium (Figure S49) is larger than that of CoP/NF (0.78 s^-1^) and NF (0.35 s^-1^).


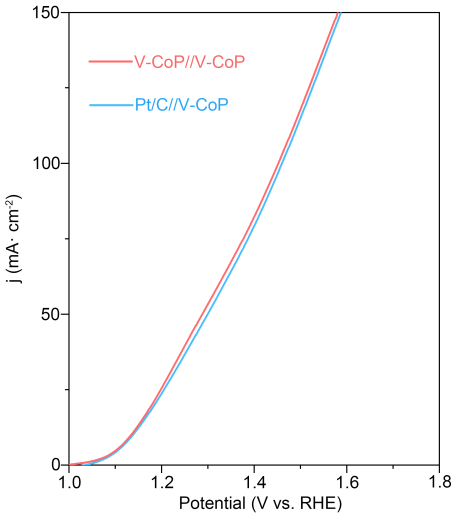


**Figure S51** Polarization curves for V-CoP//V-CoP and Pt/C//V-CoP coupling electrodes in flow-through reactor.


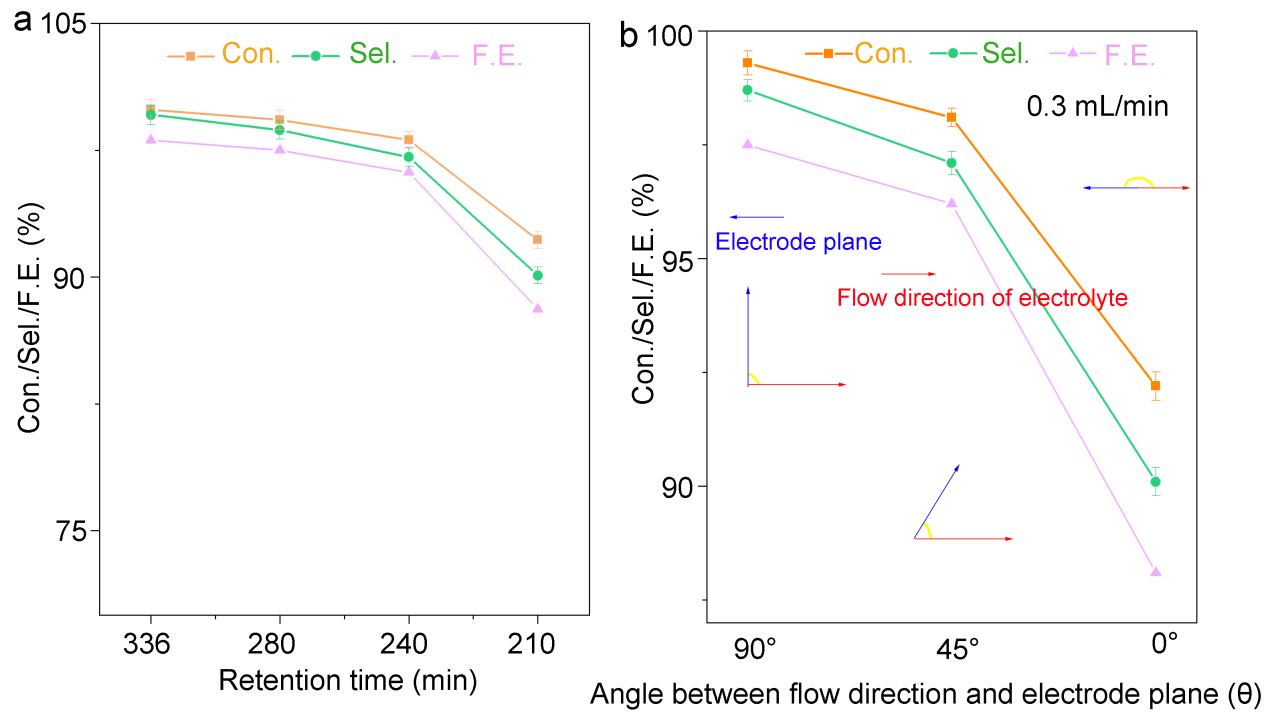


**Figure S52** (a) HMF conversion rate, FDCA selectivity, and Faraday efficiency for different retention times. (b) HMF conversion rate, FDCA selectivity, and Faraday efficiency based on the angle between electrolyte flow direction and electrode plane. The error bar represents the standard deviation of four independent measurements (n=4, mean ± SD).


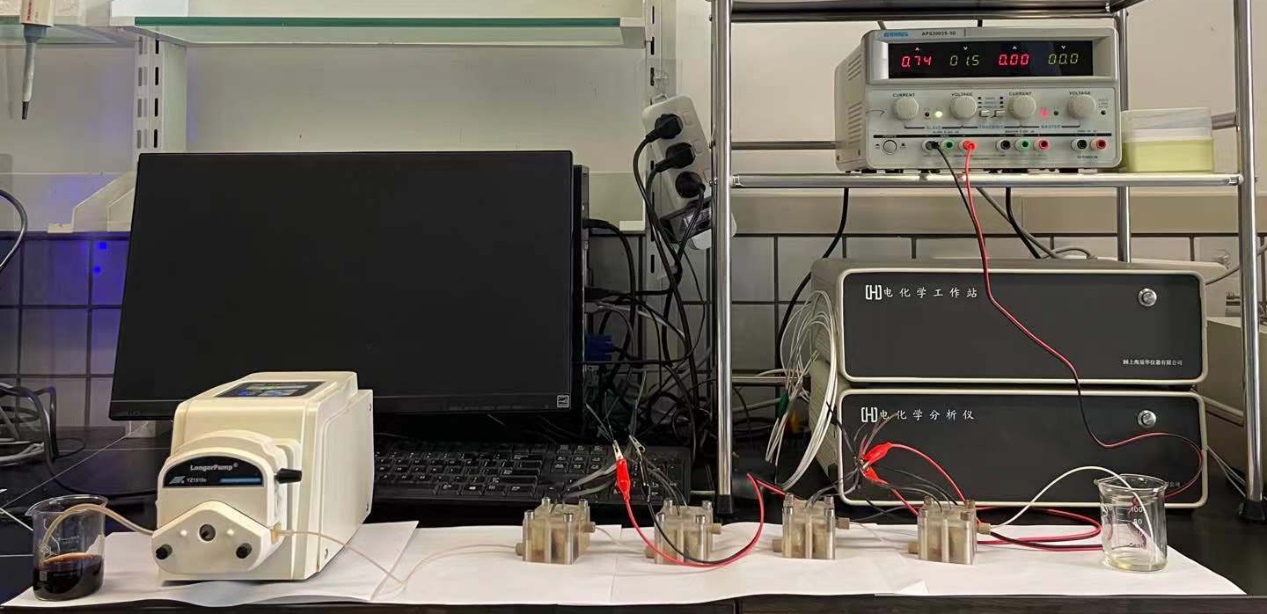


**Figure S53** The photo of HMFOR in flow-through reactor at 1.4 V direct-current power.

*
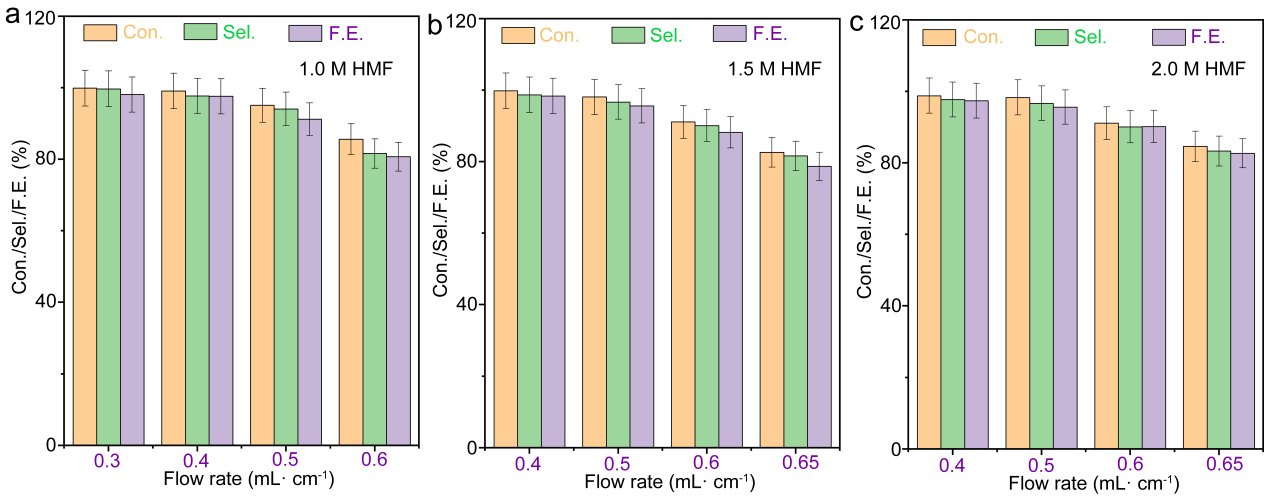
*

**Figure S54** HMF conversion rate, FDCA selectivity, and FE at different flow rates of industrial grade concentrations ((a) 1 M, (b) 1.5M, (c) 2 M) in a flow-through reactor. The error bar represents the standard deviation of four independent measurements (n=4, mean ± SD).


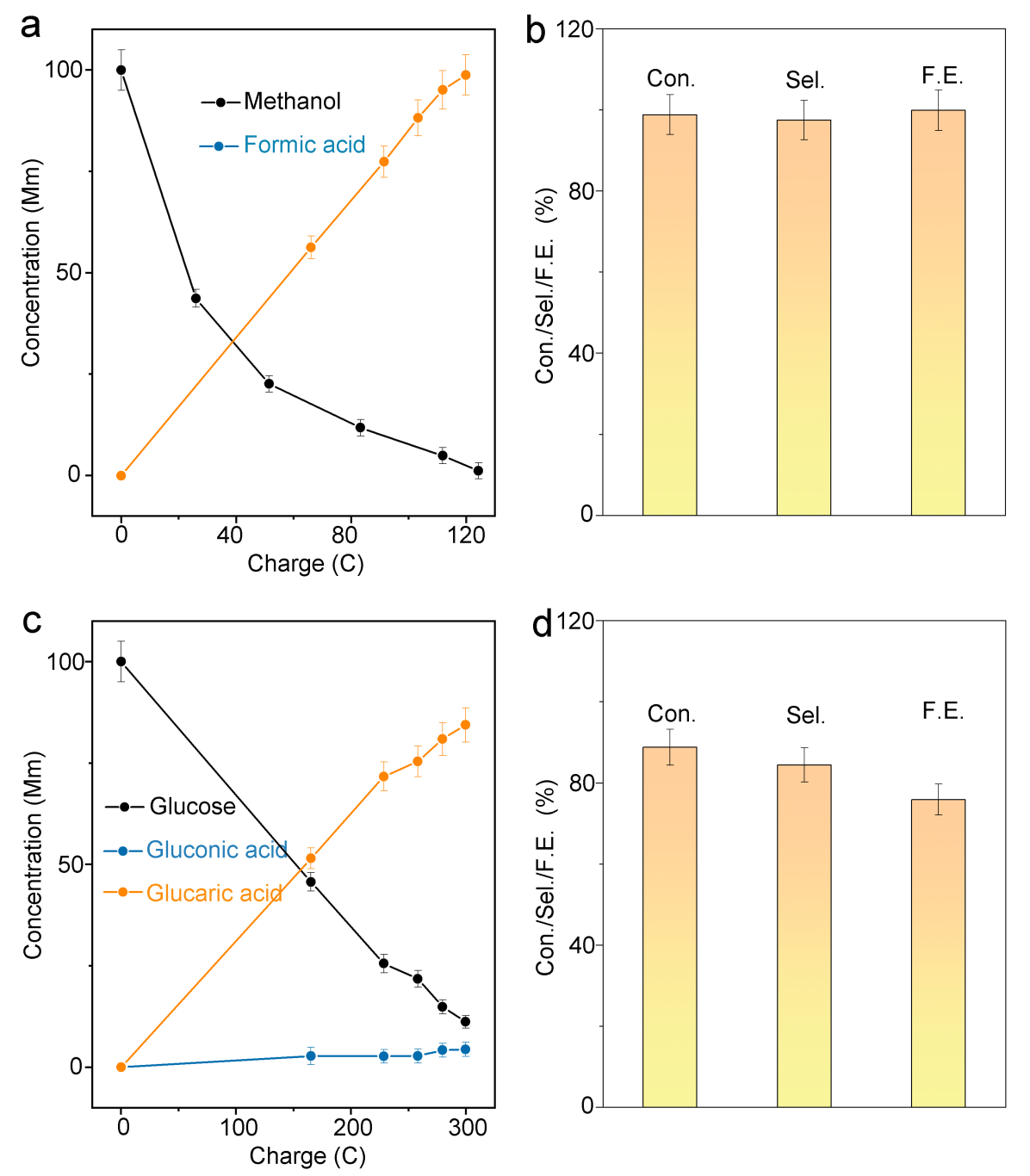


**Figure S55** (a) The concentration variation curves of methanol, formic acid, and (c) glucose, gluconic acid, and glucaric acid with increasing electricity. (b) Methanol and (d) glucose conversion, formic acid selectivity, and Faraday efficiency at V-CoP/NF electrode. The error bar represents the standard deviation of four independent measurements (n=4, mean ± SD).
